# Inter-Chromosomal Contact Networks Provide Insights into Mammalian Chromatin Organization

**DOI:** 10.1371/journal.pone.0126125

**Published:** 2015-05-11

**Authors:** Stefanie Kaufmann, Christiane Fuchs, Mariya Gonik, Ekaterina E. Khrameeva, Andrey A. Mironov, Dmitrij Frishman

**Affiliations:** 1 Department of Genome Oriented Bioinformatics, Technische Universität München, Wissenschaftszentrum Weihenstephan, Freising, Germany; 2 Helmholtz Zentrum München, German Research Center for Environmental Health, Institute of Computational Biology, Neuherberg, Germany; 3 Technical University Munich, Institute for Mathematical Sciences, Garching, Germany; 4 Institute for Stroke and Dementia Research (ISD), Klinikum der Universität München, Munich, Germany; 5 Research and Training Center on Bioinformatics, Institute for Information Transmission Problems, RAS, Moscow, Russia; 6 Department of Bioengineering and Bioinformatics, M.V. Lomonosov Moscow State University, Moscow, Russia; 7 Institute for Bioinformatics and Systems Biology, HMGU German Research Center for Environmental Health, Neuherberg, Germany; 8 Department of Bioinformatics, St Petersburg State Polytechnical University, St Petersburg, Russia; Florida State University, UNITED STATES

## Abstract

The recent advent of conformation capture techniques has provided unprecedented insights into the spatial organization of chromatin. We present a large-scale investigation of the inter-chromosomal segment and gene contact networks in embryonic stem cells of two mammalian organisms: humans and mice. Both interaction networks are characterized by a high degree of clustering of genome regions and the existence of hubs. Both genomes exhibit similar structural characteristics such as increased flexibility of certain Y chromosome regions and co-localization of centromere-proximal regions. Spatial proximity is correlated with the functional similarity of genes in both species. We also found a significant association between spatial proximity and the co-expression of genes in the human genome. The structural properties of chromatin are also species specific, including the presence of two highly interactive regions in mouse chromatin and an increased contact density on short, gene-rich human chromosomes, thereby indicating their central nuclear position. Trans-interacting segments are enriched in active marks in human and had no distinct feature profile in mouse. Thus, in contrast to interactions within individual chromosomes, the inter-chromosomal interactions in human and mouse embryonic stem cells do not appear to be conserved.

## Introduction

The experimental determination of three-dimensional structures of biological polymers is arguably one of the most direct methods for obtaining insights into their function. Structure determination has become common for some classes of molecules, particularly proteins and RNAs; however, the investigation of large-scale DNA folds using methods such as X-ray crystallography or NMR spectroscopy remains a challenging problem because of the extremely large size of chromosomes. Recent advances in high-throughput DNA sequencing have led to the emergence of an alternative experimental approach for the determination of chromatin folds: chromosome conformation capture methods [1,2] produce chromosome interaction maps that reveal intra- and inter-chromosomal contact patterns instead of precise coordinates in a three-dimensional space. These methods combine formaldehyde cross-linking of genomic regions situated close in space with fragmentation and paired-end sequencing, thereby providing insights into genome interactions in the nucleus. In particular, the Hi-C method [3] is designed to detect all pairwise interactions between chromosomal loci and has been used by many different laboratories to explore the DNA folds in multiple organisms.

The application of these new methodologies has generated a wealth of new knowledge about the spatial conformation of chromatin in eukaryotic cells. Chromosomes adopt non-random positions in the nucleus, forming the so-called chromosome territories [4]. The spatial separation of open and closed chromatin has been observed within these territories [3], and these subnuclear compartments are thought to play an important role in improving transcription efficiency, thereby representing an additional level of gene expression regulation.

The folding structure of interphase chromosomes is thought to be hierarchical [3,5]: according to the fractal globule model each chromosome strand collapses into small globular structures, which in turn collapse into globular folds. This hierarchical arrangement facilitates the efficient use of the limited space within the nucleus during the interphase.

The fractal globule model was experimentally confirmed in recent studies of the intra-chromosomal folding properties of human and mouse interphase chromosomes [6]. According to these studies, the chromosomes are organized into topological domains with large linear regions that have a high number of contacts within them and depletion of contacts between them. The borders of these regions are well defined and enriched with classical insulator features such as CCTC-binding factor (CTCF). The structure of the topological domains indicates significant evolutionary conservation between human and mouse chromosomes, which is assumed to be of high functional importance.

Previous research has shown that there is a strong connection between spatial chromatin organization and gene regulation. For example, the promoter-enhancer contacts established in fibroblasts by chromatin looping are relatively stable [7]. Khrameeva et al. [8] demonstrated that spatial proximity is correlated with co-expression and the functional similarity of genes in human fibroblasts.

Previous research in this area has mostly focused on the folding of individual chromosomes, whereas inter-chromosomal contact networks have received much less attention, particularly in mammals [8]. As the signal-to-noise ratio is usually lower in inter-chromosomal interactions and methodological biases have stronger effects, these types of contacts have to be handled very carefully and the results should be considered with caution. In addition, a holistic comparison of the chromatin interaction maps between different species has not yet been performed. Thus, the identification of common structural properties between species as distant as humans and mice could provide new insights into the functional importance of contacts between different chromosomes. In principle, this comparison could be made on the basis of Hi-C matrices containing normalized read counts in each cell. However, methodologically, it is much easier to interpret binary contact matrices that only contain the information about whether there are probable contacts between genomic segments on the basis of a certain cutoff for the interaction probabilities. These binary contact matrices can be transformed into networks, which are easy to handle and facilitate the simple detection of structural features. In addition to providing an overall view of the structure and topology of the interactome, this type of network representation is highly suitable for identifying important structural properties of the three-dimensional DNA arrangement. For example, it can be used to detect highly connected segments and genes (network hubs) as well as for investigating functionally related substructures.

In the present study, we aimed to utilize the network approach to identify new characteristics of the chromatin interactomes in two mammalian species: humans and mice. We performed a detailed analysis of the inter-chromosomal interactome, where we used a mathematical approach to transform Hi-C data into a network, while removing experimental biases during normalization, as proposed by Kruse et al [9]. We were particularly interested in long-range gene–gene contacts, which may indicate the existence of transcription factory-style spatial clusters. Previously, Kruse et al. [9] created a gene interaction network (GIN) for *Saccaromyces cerevisiae* and demonstrated the significant co-localization of genes and the clustering of replication origins. We adapted their approach to a lower read coverage and the more complex chromosome structure of larger mammalian genomes, i.e. humans and mice.

In this study, we investigated inter-chromosomal segment and gene–gene contact networks (also: trans-interaction networks) using embryonic stem cells (ESCs) on the basis of Hi-C data obtained by Dixon et al [6]. We found that both networks share certain properties such as the power-law degree distribution and strong clustering, and we determined some of their unique characteristics such as highly flexible genome regions in mouse chromosomes. In agreement with previous research, we provide evidence that human trans-interacting segments are enriched with active marks and that co-expression and functional similarity are correlated with spatial proximity.

## Materials and Methods

### Hi-C data and statistical confidence of inter-chromosomal interactions

We used experimental Hi-C data for *Homo sapiens* (assembly hg18) and *Mus musculus* (assembly mm9), which were recently published by Dixon et al. [6] (GEO: GSE35156). To facilitate better comparability, we only used ESC data from both species. We normalized the raw Hi-C data with *hicpipe* (version 0.93), as described by Yaffe and Tanay [10], and used the resulting parameters to calculate the background interaction probability.

Hi-C-derived DNA–DNA contact data are known to exhibit systematic biases such as unspecific ligation of fragments as well as varying fragment lengths, GC content, read mappability or uniqueness and restriction enzyme site density. *Hicpipe* allows to estimate the contact probability of two reads based on these biases, so that for example reads that map to multiple regions in the genome can be excluded. To assess the statistical significance of fragment interactions and to filter out unspecific contacts in yeast, Kruse et al. [9] calculated the confidence of a measured fragment pair contact using a binomial distribution based *p*-value, followed by false discovery rate control. Because of the larger size and more complex multi-chromosome structure of mammalian genomes compared with that of *S*. *cerevisiae* genomes, we had to make some adjustments to their method to account for the resulting lower read coverage per fragment pair in the human and mouse genomes. To capture sufficient signals we binned DNA fragments into 500 kb segments, which made the read coverage sufficient to calculate the average interaction probabilities on the basis of the normalization results from *hicpipe* for each pair of segments. This relatively low-resolution binning is necessary, because read coverage is not sufficient for binning into smaller segments. As a consequence, our analyses are coarse compared to those of smaller genomes. We found that for smaller window sizes the read density is too low for contact network construction. At the same time, larger window sizes (up to 2 Mb), while producing similar overall structures, are not detailed enough.

Similar to previous research [9, 11], we used the binomial distribution to estimate the *p*-values that reflect contact significance separately for each pair of chromosomes:

$$P(bin_{a},bin_{b})=\sum_{i=k}^{n}\left(\begin{array}{c} n\\ k \end{array}\right)m_{norm}^{i}{\left(1-m_{norm}\right)}^{n-1}$$

Where *bin*_*a*_,*bin*_*b*_ are two 500 kb bins, *m*_*norm*_ is the average interaction probability of all pairs of fragments from *bin*_*a*_ and *bin*_*b*_, *k* is the observed summarized number of reads in *bin*_*a*_,*bin*_*b*_, and *n* is the total number of observed reads for the two chromosomes from which *bin*_*a*_,*bin*_*b*_ stem. As such, the p-value describes the probability that an interaction of two bins is observed at least as many times as our data suggests, given the background probability reflecting Hi-C biases.

R version 3.0.0 [12] was used for false discovery rate control according to Benjamini and Hochberg [13], using the R function *p*.*adjust* (method “fdr”) to calculate confidence values (q-values), for the contacts. Because the calculation of contact *p*-values is directly dependent on the total number of observed contacts and because we calculated these values separately for each pair of chromosomes, the *p*-values computed for smaller chromosomes, which possessed fewer interactions, tended to be lower than those computed for large chromosomes. To remove this bias, we normalized the q-values against the chromosome length.

### SIN and GIN

Using a q-value-based confidence cutoff we created binary contact maps for 500 kb genome segments, which were transformed into inter-chromosomal segment interaction networks (SINs) by considering segments as nodes and connecting them by edges if they shared a confident contact and resided on different chromosomes. Furthermore, we downloaded human and mouse genes from ENSEMBL BioMart (version 72) [14] and mapped their coordinates to version hg18 and mm9 of the human and mouse genomes, respectively, using liftover [15]. Each gene was then assigned to the 500 kb DNA segment where most of it was located. A gene interaction network (GIN) was created for each species by connecting genes (nodes) by edges if their corresponding segments were in contact according to SIN. This approach clearly tends to overestimate the number of gene interactions since all genes in a 500 kb region are assumed to interact with all genes in a contacting 500 kb region, though this might be true only for a subset of genes. At the current sequencing depth, a more refined mapping is only possible for smaller genomes. We thus focus our analyses on the SINs.

### Randomization of SIN and GIN

Randomization was performed at the level of segment interactions. In SIN, each node represented a 500 kb segment and each edge indicated a physical contact according to the filtered interaction matrix. We used the approach described by Witten and Noble [16] to create a basis random network by randomly distributing segments in a unicube and drawing edges between the *x* closest pairs according to the Euclidean distance, where *x* is the number of edges present in the original network. Following Kruse et al. [9], we permuted the set of edges (E) *10 x |E|* times and then exchanged edges between neighbors if the exchange led to a higher transitivity in the random network, thereby obtaining a better approximation of the clustering behavior of the original network. Following this, we transformed the randomized SIN into a gene contact network by randomly drawing *y* genes without replacement for each segment, where *y* is the number of genes originally residing in this segment.

This randomization procedure is based on an artificial three-dimensional structure and then modified to better mirror the original network’s properties. We believe that this methodology is superior over simpler randomization approaches, such as contact shuffling, as it provides a more realistic background model.

### Network analysis

We visualized the networks using Cytoscape [17] and employed its analysis tools to calculate the following basic network statistics:

#### Clustering coefficient

The clustering coefficient describes the degree to which the nodes in a network tend to cluster together, which is calculated for each node as

$$C_{n}=\frac{2e_{n}}{\left(k_{n}(k_{n}-1)\right)}$$

where *n*, *e*_*n*_, and *k*_*n*_ is a node in the network, the number of connected pairs between all neighbors of *n* and the number of *n*’s neighbors, respectively. A network’s clustering coefficient is the average clustering coefficient of all nodes, where for nodes with less than two neighbors, *C*_*n*_ *= 0* to avoid overestimation of clustering in the presence of many singletons.

#### Characteristic path length

The average length of the shortest paths between all pairs of nodes.

#### Connectivity centralization [18]

The connectivity centralization provides a measure of the degree distribution, which is calculated as

$$Centralization=\frac{N}{N-2}\left(\frac{\text{max}(k)}{N-1}-\frac{mean(k)}{n-1}\right)\approx \frac{\text{max}(k)}{N}-\frac{mean(k)}{n-1}$$

where *max(k)* is the highest degree in the network, *mean(k)* is the average degree, and *N* is the number of nodes.

#### Diameter

The longest shortest path between any two nodes in the network

#### Average degree

The average number of neighbors per node.

#### Heterogeneity [18]

Heterogeneity as defined by Dong and Horvath [18] reflects the variation of node degrees in a particular network

$$Heterogeneity=\frac{\sqrt{\text{var}\;iance(k)}}{mean(k)}$$

#### Isolated nodes

Isolated nodes are those with a degree of zero.

### Feature composition of connected segments

We calculated the overlap of trans-interacting segments, i.e., contacts between segments from different chromosomes, and non-trans-interacting segments using a broad set of genomic features, including the coverage of lamina-associated domains (LAD), repeat coverage (LINE, SINE, and LTR), replication timing domains, histone modifications (H3k4me3, H3k4me1, and H3k27ac), open chromatin and DNase I hypersensitivity sites (S1 Table). For this analysis, we excluded sex chromosomes to avoid a bias towards inactive marks. In addition, we calculated the average number of binding sites for 55 transcription factors described by the ENCODE project [19] in trans-interacting and non-interacting segments. It has to be noted that we were unable to find feature data from cell lines that are directly matching the cell lines used for the Hi-C data. In these cases we used cell lines that are as comparable as possible to get a good approximation.

### Spatial (gene) clusters

We defined spatial gene clusters as all genes from two interacting segments that were present in the normalized and filtered SIN. We downloaded the gene positions from ENSEMBL (version 72) [14]. Using ENCODE [19] data for 55 transcription factor-binding sites, we analyzed the percentage of genes in each spatial cluster with a binding site for any of these transcription factors.

### Investigation of contacts between HOX clusters

HOX genes are involved in embryonic development and are organized into linear clusters of genes in both the human and mouse genomes [19,20]. Because the expression of these genes is directly dependent on their linear order, spatial co-localization could theoretically expand this feature by combining multiple clusters in the third dimension. Therefore, we investigated the potential spatial contacts between HOX clusters in both species by calculating interaction confidence as described above for the entire HOX cluster regions instead of for 500 kb segments.

### Analysis of the correlation between spatial proximity and functional properties of genes

We aimed to identify the possible interaction between the spatial proximity of chromosomal regions and the functional properties of genes residing in these regions using two alternative approaches. First, we calculated Pearson’s correlation coefficients for the values of all inter-chromosomal segment pairs and measures of the functional similarity of genes (gene ontology (GO) term similarity and co-expression, as described in the following two sections).

The second approach, which was applied previously by Khrameeva et al. [8] to human fibroblast Hi-C data [3], was utilized to consider the large amount of noise present in Hi-C data, which is caused by the random Brownian motion of chromosomes, averaging over millions of cells, and possible experimental biases. We converted the data into *N* tuples of *(x*,*y)*, where *x* is the spatial proximity value of a segment pair and *y* is the corresponding co-expression measure or GO term similarity. We binned the data as follows:

1. We ranked all tuples *(x*,*y)* according to *x*
2. We introduced 30 bins, each of which held *N/30* entries. We equally distributed the entries in the bins according to their rank.
3. We represented each bin by *mean(x)* and *mean(y)*

Binning is known to overestimate the value of true correlation due to variance reduction [21,22]. Therefore, we performed the binning procedure described above using 1000 datasets, where pairs of *x* and *y* were randomly selected and *p*-values were calculated to assess the significance of the association, but without taking the correlation coefficient as an indicator of the correlation strength. The significance of the result was assessed on the basis of the cumulative distribution function.

### GO enrichment analysis

The GO term similarity between the genes within spatial clusters was calculated using the Bioconductor package GOSemSim [23], using Wang’s method and *rcmax*.*avg* for summarization. We ran the method for each GO hierarchy (Molecular Function, Biological Process, Cellular Component) and combined the results by calculating the average similarity score for each segment pair, where we only considered the hierarchies that were available for the segments. We determined the correlations between the GO term similarity and spatial proximity values of the inter-chromosomal all-*vs*.-all set of 500 kb segments by the two approaches described above.

### Correlation between the physical co-localization and co-expression of genes

Using expression data for 24,115 genes in 43 hESC samples [24] (see original publication for complete list of samples), we calculated combined co-expression values (S1 Methods), as described by Khrameeva et al. [8], and we determined their association with the spatial proximity values (see S1 Methods). No comparable data were available for mouse ESCs; therefore, we only performed this analysis for human ESCs.

### Network comparison

We compared the human and mouse GINs (HGIN and MGIN, respectively) on the basis of the genomic regions with conserved gene order in these two species, which were detected using SyntenyMapper [25]. For each region with a conserved gene order in the human genome, we transferred the contacts of all genes situated in this region to their mouse orthologs using SyntenyMapper, thereby yielding the mouse gene set, *H*_*M*_. Thus, the contacts between mouse genes in *H*_*M*_ were inferred from the human experimental data by ortholog mapping. In addition, we utilized the experimentally measured contacts between genes within the region of the mouse genome that was equivalent to the human region to obtain the set of interacting genes, *M*. The overlap between *M* and *H*_*M*_ was normalized on the basis of the size of the smaller set of connected genes and it was then used as a measure of network similarity (S1 Fig).

## Results

### The mouse inter-chromosomal interactome is strongly shaped by two highly connected segments

**Fig 1:**
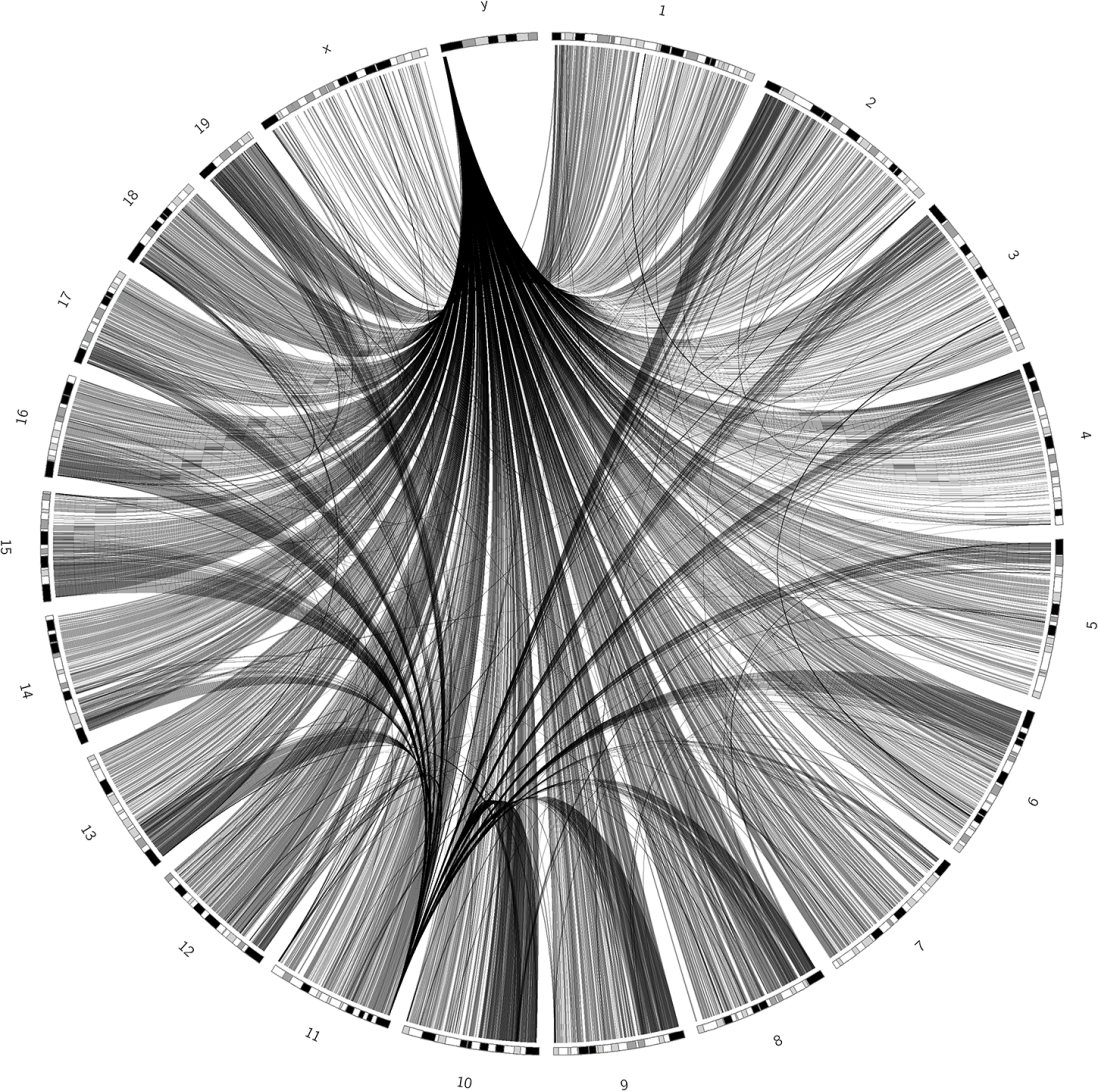
Circos visualization of the segment interaction network in *M*. *musculus* with a q-value cutoff of 1e-6. The banded ideograms are chromosomes and the black lines between them imply high-confidence contacts. On chromosome Y, a highly interactive, gene-free region makes contacts with almost the entire genome. Another high-contact region on chromosome 11 preferentially interacts with centromere regions at the chromosome ends.

The mouse SIN is densely connected even with strict q-value cutoffs, with a low percentage of unconnected segments (32.2% with a q-value cutoff of 1e-6) (Fig 1, Table 1). The overall network structure differs dramatically from the randomized SIN (RSIN, see S2 Fig), where the contacts are uniformly distributed among the segments. The most highly connected region is situated on chromosome Y, where the gene-less segment from 2,500,000 bp to 3,000,000 bp forms the most contacts with other segments in the whole network, with a degree of 3,152 (with the same cutoff as that used above), which involves 70% of all the edges in this network. Another region with a high number of contacts is located close to the telomere of chromosome 11 (between positions 3,000,000 bp and 3,500,000 bp), which has contacts with 979 other segments on all chromosomes. Interestingly, the latter segment preferentially makes contacts with segments located proximal to the telomeres of other chromosomes. Because the mouse genome is telocentric, this may imply the existence of a centromeric cluster with chromosome 11 at the center.

**Table 1:** Sizes of the segment interaction networks and their corresponding largest connected components for human (HSIN) and mouse (MSIN) genomes, and their randomized versions (RHSIN for human and RMSIN for mouse, respectively).

	HSIN	RHSIN	MSIN	RMSIN
Q-VALUE THRESHOLD	1E-3	1E-3	1E-6	1E-6
**#NODES**	5,732	5,732	5,093	5,093
**#CONNECTED NODES**	2,500 (43.61%)	4,531 (79.05%)	3,450 (67.74%)	4,211 (82.68%)
**#EDGES**	4,520	4,517	4,483	4,485
**LARGEST COMPONENT: #NODES**	2,349	3,601	3,282	3,664
**LARGEST COMPONENT: #EDGES**	4,435 (98.12%)	3,894 (86.21%)	4,171 (99.67%)	4,147 (92.46%)
**2** ^**ND**^ **LARGEST COMPONENT: #NODES**	5	14	4	17

The largest components of the SINs contain the majority of the genes.

The two highly connected regions on chromosomes 11 and Y, as described above, had observed read counts that were 5.45 and 4.54-times higher than the average, respectively, and they remained highly interactive after normalization with respect to known Hi-C biases.

However, neither of these two highly interactive segments could be in contact with so many other chromosomal regions simultaneously. In contrast to single-cell Hi-C, the Hi-C data reported by Dixon et al. [6] were measured using many different cells of the same type. Thus, a contact between segment A and segment B does not mean that this interaction occurs in all cells; rather, it could have occurred in any of the cells tested in the experiment. Therefore, the highly interactive regions such as those on chromosomes 11 and Y are expected to form contacts with different segments at different time points and in different cells.

### Short human chromosomes participate in more trans-interactions than long chromosomes

**Fig 2:**
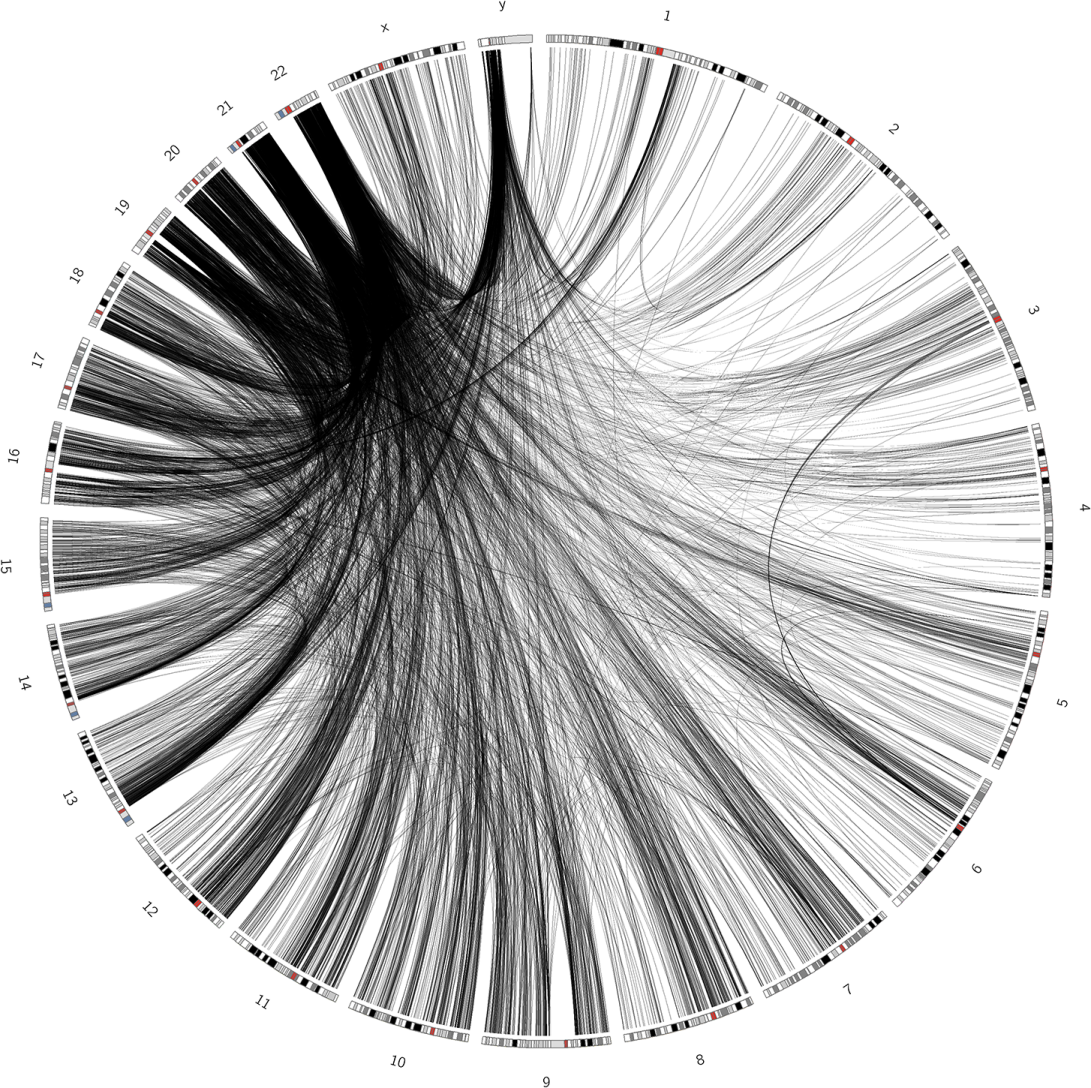
Circos visualization of the human segment interaction network with a q-value cutoff of 1e-3. The majority of the contacts are formed between short chromosomes or from short chromosomes to others. Red bands mark centromeres.

**Fig 3:**
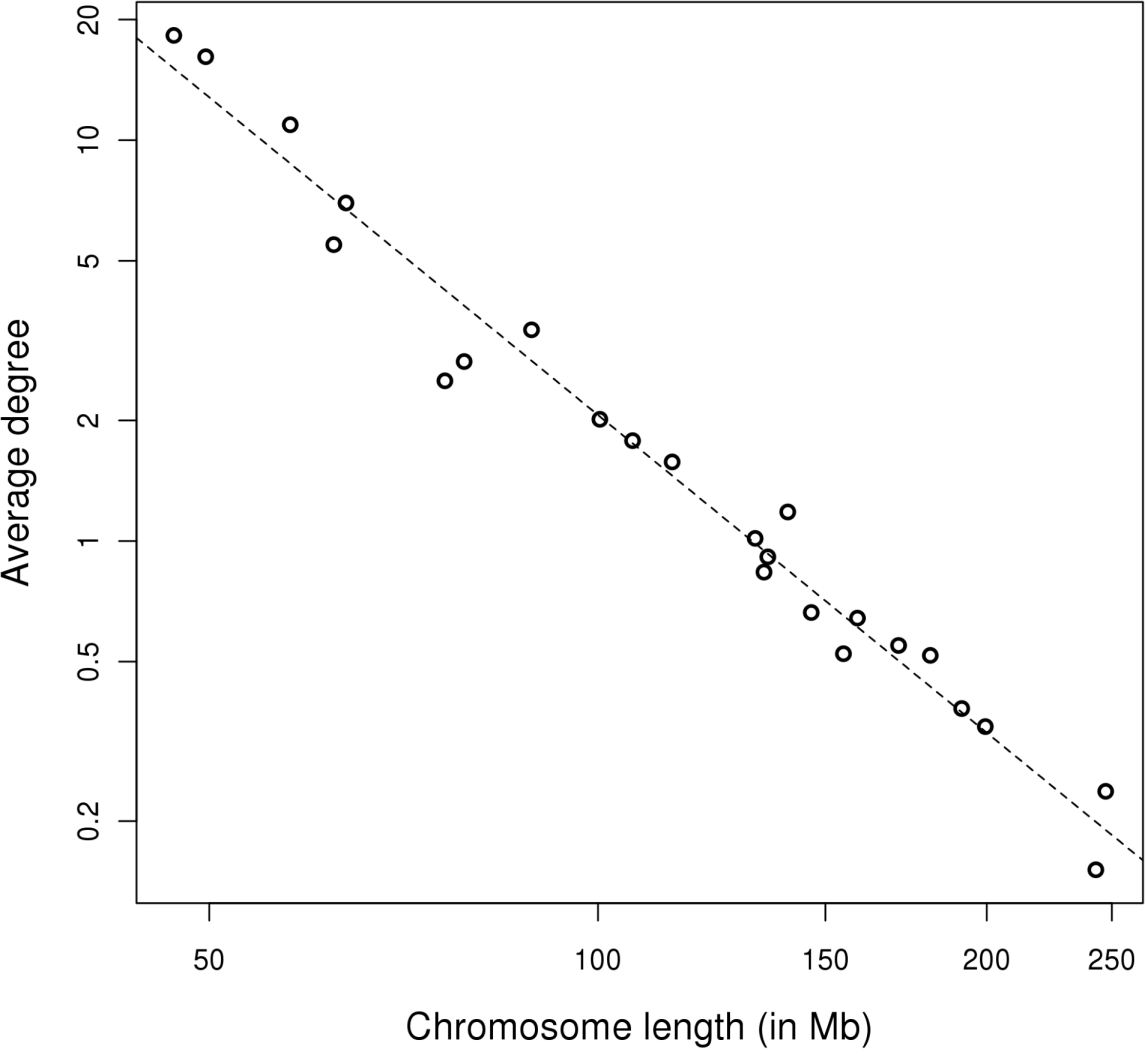
Relationship between the average segment degree, i.e. contact number, and chromosome length in the human genome. There is a clear linear correlation on the log–log scale (Pearson correlation coefficient = −0.70), which illustrates the high density of connections between short chromosomes.

The landscape of human inter-chromosomal contacts also differs greatly from the corresponding randomized network (Fig 2 and S3 Fig); however, in contrast to the mouse SIN, no obvious single segments were involved in the majority of the contacts. In some chromosomes (1 to 3, 5 to 8, 11 to 13, X) the contacts occur more frequently close to their centromeres. Shorter human chromosomes form more interactions between themselves and with long chromosomes than the latter form between themselves. Indeed, there is a clear inverse correlation between the average number of contacts per segment and chromosome length (Fig 3, Pearson’s correlation coefficient = −0.70). In mice, a similar correlation was obtained, with a slightly greater deviation from the regression line if chromosomes 11 and Y were excluded, which harbor the highly interactive segments described above (S4 Fig, Pearson’s correlation coefficient = −0.87).

Similar to mice, the human Y chromosome has a high number of contacts, although this effect was less pronounced. On average, the chromosomes are involved in 376.67 contacts and the Y chromosome forms 481 connections, despite its short length.

### Comparison of human and mouse interactomes

We generated MSINs and HSINs using q-value cutoffs that ranged from 0.05 to 1e-8 (S2 Table). The q-value distributions differ in both species, thereby yielding different degrees of connectivity with the same cutoffs and necessitating the use of different q-value thresholds for these two species to ensure comparability. This is mainly due to differences in genome size and read coverage, which is almost twice as high in mouse as in human (average read coverage on 500kb segments in human is 2.53, in mouse 4.25), while background contact probability is in a similar range. Assuming a similar or a slightly smaller (due to the smaller genome) degree of inter-chromosomal connectivity for mouse, this higher read coverage is probably caused by experimental artefacts leading to a higher amount of noise. We thus use a stricter q-value filter to be able to compare data between human and mouse. Additionally, we investigated the robustness of the network with respect to this parameter and found that the overall network structures were highly similar for the majority of q-value thresholds.

The number of connected nodes and edges rapidly declines as the cutoff decreases in the human genome, whereas this relationship is less pronounced in the mouse genome. Given the structural properties of the two networks described in the previous sections, we speculate that this difference is caused by a slight difference in the spatial genome organization of the two species. It is known that the centromeres in human ESCs (hESCs) are less likely to localize to the periphery and the nucleolus than those in differentiated human cells [26], implying that centromere co-localization is less pronounced in this undifferentiated cell type. Similarly, centromere clustering was reported to be more often in differentiated mouse cells [27]. However, there has been no previous global comparison of the chromatin interactomes in human and mouse ESCs on the basis of Hi-C data. The gene-rich human chromosomes are co-localized in the nuclear center [28]; however, no similar observations have been made in mice.

According to our data, the human genome tends to form a high percentage of its contacts with at least one short and often gene-rich chromosome. The centromeric regions of long human chromosomes also tend to be highly interactive. Similarly, the centromeres in the mouse genome frequently appear to co-localize with a centromeric region on chromosome 11. A region on the Y chromosome also forms an extremely high number of contacts with segments throughout the mouse genome with very high confidence values, thereby suggesting the high flexibility of this region. This extreme behavior is not observed in the human genome; however, we found that the human Y chromosome was in contact with more other segments than the average chromosome, despite its short size.

For more in-depth analysis, we selected q-value thresholds of 1e-3 and 1e-6 for the human and mouse SINs, respectively. Using these cutoffs, the two networks exhibit comparable connectivity where they contain 4,520 and 4,483 edges, respectively (Table 1). Table 2 summarizes the basic network properties of the HSIN, the MSIN, and their randomized versions using the selected cutoffs. The basic network statistics reflects the structural differences in the networks. The extraordinarily high degree of the mouse Y chromosomal segment and the resulting hub-based network topology yield a low characteristic path length, high centralization, and low diameter compared with HSIN, although the average degree is in the same range. The network heterogeneity is the property that differs the most in the two species (2.7 in humans vs. 26.3 in mice). The network heterogeneity measures the similarity of nodes with respect to their degree; thus, the hub with many thousands of contacts in mice again leads to a very high value. Another parameter that measures the network variance and its conformity to the Poisson distribution is variance divided by mean, which also differs significantly between HSIN and MSIN (1215.3 vs. 11.4). This measure is close to 1 in both randomized networks, indicating that, in contrast to the original networks, these are in fact very uniform.

**Table 2:** Basic network properties of the human segment interaction network (HSIN) and its randomized version (RHSIN) as well as the mouse segment interaction network (MSIN) and the randomized version (RMSIN).

	HSIN	RHSIN	MSIN	RMSIN
**CLUSTERING COEFFICIENT**	0.006	0	0.166	0
**NETWORK DIAMETER**	13	40	7	31
**NETWORK CENTRALIZATION**	0.014	0.001	0.619	0.001
**CHARACTERISTIC PATH LENGTH**	4.690	16.066	2.137	13.177
**AVERAGE DEGREE**	1.577	1.574	1.761	1.761
**NETWORK HETEROGENEITY**	2.695	0.805	26.259	0.759
**VARIANCE/MEAN**	11.44	1.023	1,215.34	1.016
**ISOLATED NODES**	3,232	1,201	1,641	882

Irrespective of the overall structural differences, both human and mouse genomes exhibit a high degree of inter-connectivity and chromosomal clustering, where the network topology is dominated by a few highly interactive segments (S5 and S6 Figs), with most segments exhibiting low contact numbers. Similar results have been reported for the strongly clustered yeast inter-chromosomal interaction network [9].

We created the randomized networks (RSINs, S3 Table) to ensure the maintenance of the clustering coefficient and the transitivity of the original networks; however, mouse and human RSINs still possessed some common characteristics. Both of them lacked evident clusters of contacts, thereby leading to a higher network diameter, lower centralization, and higher path length with fewer singletons. The contact patterns were much more homogeneous than those in real networks, where most segments participated in a similar number of interactions (S2 and S3 Figs). Thus, the strong clustering of centromeric regions, the central role of the Y chromosome in the mouse genome, and the apparent co-localization of short chromosomes in the human genome are non-random effects.

### Spatial connectivity and genomic features

We divided 500 kb human and mouse genome segments into those that formed inter-chromosomal contacts and those that did not, and we analyzed the overlaps among the connected and unconnected segments of the autosomes with the genomic features listed in S2 Table. It has been reported that active marks in the human genome are correlated with the enrichment of trans-chromosomal contacts [3,29]. Our results confirm this tendency for the H3k4me3, H3k4me1, H3k27ac, H3k9ac and H3k36me3 marks obtained from comprehensive ENCODE datasets (S4 Table). Although the frequencies of these five marks greatly differ in the human genome (2.5%, 15.5%, 4.4%, 4.8%, and 17.7% of the base pairs in the human genome are covered by the peaks of these marks, respectively), they are >14% more frequent within the trans-interacting segments. The experimental data on H3k27ac in the human genome corresponds to a differentiated cell type; however, it exhibits a very similar enrichment rate to other data.

Based on ENCODE data, the frequencies of all five types of histone modifications are lower in the mouse genome than in the human genome (2.1%, 6.6%, 2.2%, 2.3%, and 4.7% for H3k4me3, H3k4me1, H3k27ac, H3k9ac and H3k36me3, respectively). However, irrespective of the differences in the total coverage, the active histone marks are neither enriched nor depleted in the mouse trans-interacting segments.

**Fig 4:**
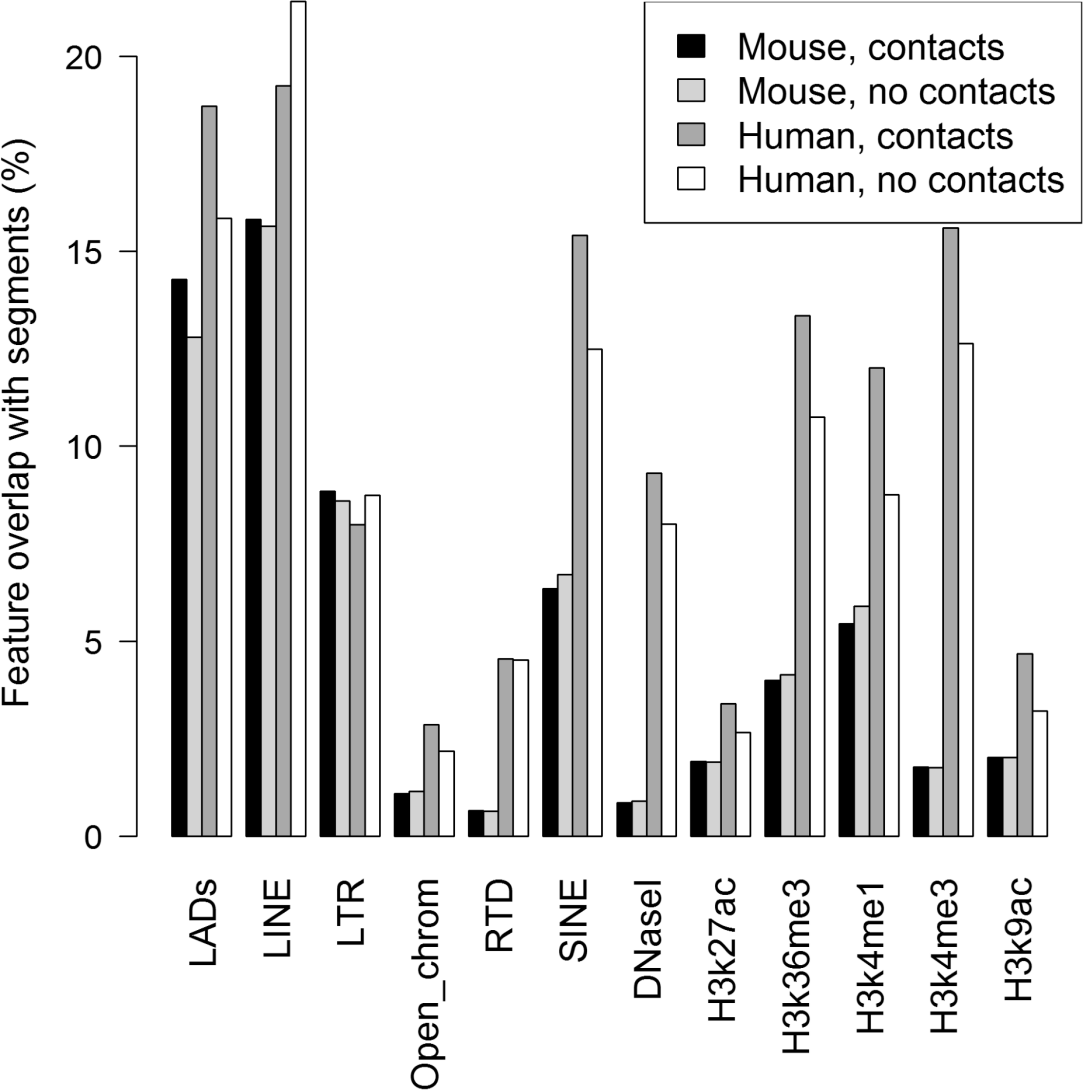
Enrichment/depletion of human and mouse genomic features in trans-interacting segments *vs*. non-trans-interacting segments. Results are given as percentage of base pair overlap.

In the human genome, the trans-interacting segments match with the active histone marks, open chromatin, gene-rich areas (47.13% of the trans-interacting segments and only 40.98% of other regions overlapped with genes), and SINE repeats (Fig 4), which are also correlated with euchromatin. The repressive marks LINE and LTR are depleted, whereas the other heterochromatic feature, LADs, is enriched. The replication timing domains overlap to a similar extent with trans-interacting and non-interacting segments. In contrast, the mouse trans-interacting segments behaved very similarly to other segments. Only LADs are slightly enriched in trans-interacting segments, similar to human.

These results imply that trans-interacting segments in humans are mostly euchromatic, whereas those in mice are not more active than other segments. Instead, they show a similar feature profile than segments without contacts, possibly implying that the mESCs were in a slightly different differentiation phase than the hESCs used in the experiments.

After analyzing the average number of transcription factor binding sites in the trans-interacting segments compared with those in other segments, we found that the highest increases were in the number of binding sites for CTCF (10.91 sites *vs*. 8.28) and RAD21, a subunit of cohesin (15.64 sites *vs*. 11.27) (S5 Table). Both these transcription factors are known to be involved in the spatial organization of chromatin.

### Generation of GINs

We built GINs on the basis of the previously discussed SINs by introducing edges between all pairs of genes from two segments with contacts. Because the mouse SIN was greatly affected by a gene-less segment on chromosome Y, its overall structure and connectivity changed when it was transformed into GIN. Thus, we used networks where a comparable amount of genes participated in at least one contact, which we obtained using a confidence cutoff of 1e-4 in both the human (30.05% of genes, S6 Table) and mouse (29.29% of genes) genomes.

Using the selected confidence cutoff, human GIN (HGIN) and mouse GIN (MGIN) exhibit relatively low connectivity (clustering coefficients, human = 0.001, mouse = 0.009). Even without the large number of contacts with the gene-less segment on Y, the MGIN contains around 1.5 times the number of edges found in the HGIN (66,331 edges vs. 41,483 edges, Table 3). However, because the number of connected nodes is similar (6,078 vs. 6,543), the MGIN is more densely connected.

**Table 3:** Comparison of the size and connectivity of the human and mouse gene interaction networks using a q-value cutoff value of 1E-4.

	*H*. *sapiens*	*M*. *musculus*
**#SEGMENTS**	1,869	1,446
**#GENES PER SEGMENT**	5.45	5.56
**#EDGES**	110,433	99,811
**#NODES**	20,229	22,341
**#SINGLETONS**	9,946 (49.17%)	14,297 (63.99%)

The higher connectivity leads to a lower average path length between any two nodes in the mouse network. However, although degrees over 100 are more common in the MGIN, the degree distribution in both species follows a power-law distribution, which was also reported for the yeast GIN described by Kruse et al. [9].

SINs exhibit a strong clustering behavior in both species, where most segments are either part of a large spatial cluster or have no connections at all. Some edges of SIN are deleted during the transformation into GIN if at least one of the nodes with which they have connections is a segment without any genes. This decomposes GINs into a higher number of connected components, although the core cluster that contains approximately 90% of all edges is still maintained.

### Binning approach unveils hidden correlations

We also investigated the relationship between the co-expression or GO term similarity and spatial proximity. We were unable to find any correlations in the raw data, where the Pearson correlation coefficients were all very close to zero and ranged among −0.03 (GO term similarity, human), 0.03 (co-expression, human) and 0.09 (GO term similarity, mouse). However, after applying the binning procedure described in the Methods, correlations with the mean values were not only observable, but in fact very strong and statistically significant when compared to randomized data. A more detailed description is provided in the following sections.

### Functional similarity between co-localized genes

**Fig 5:**
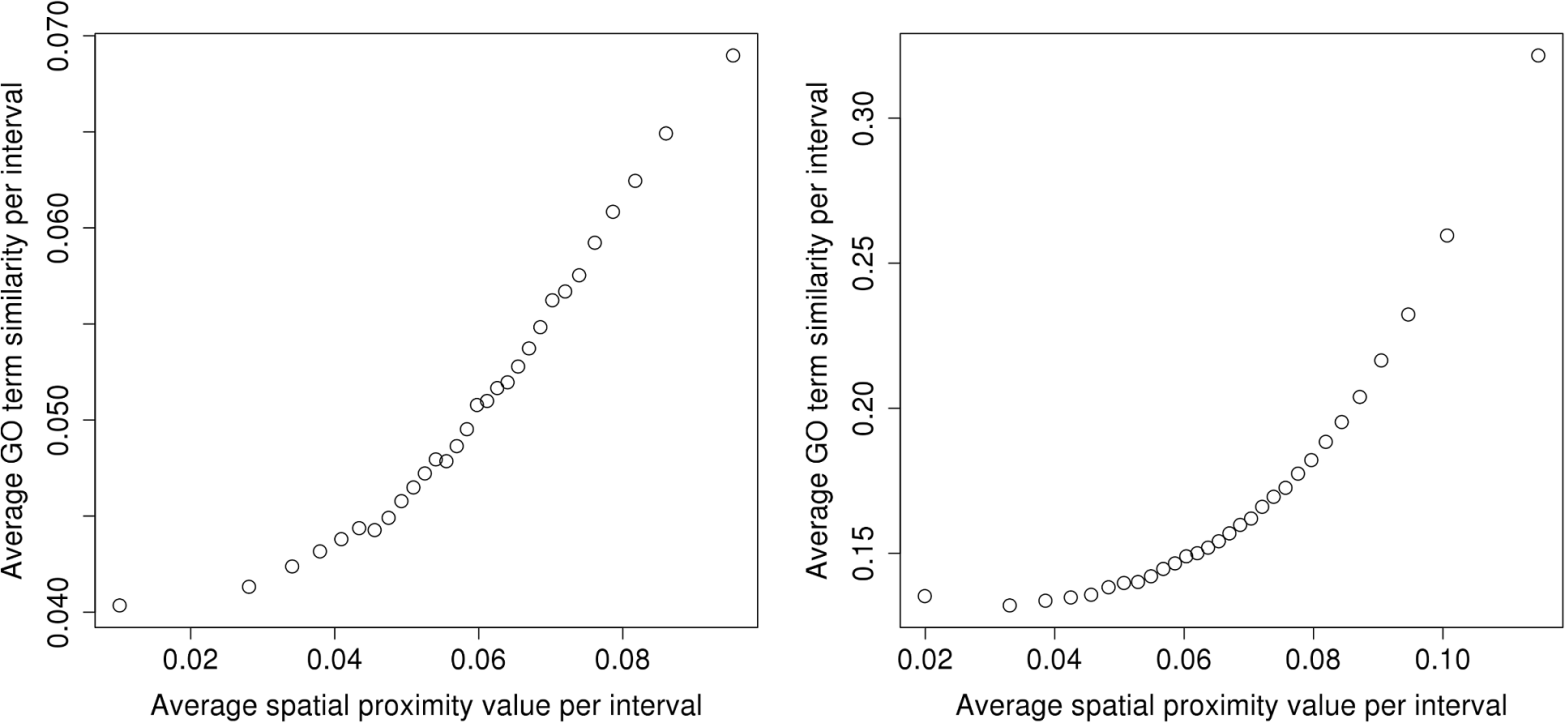
Strong correlation between mean GO term similarity and mean spatial proximity in human (left) and mouse (right) genomes. Data were binned into 30 intervals according to the spatial proximity value (see Methods for details). The observed correlations are statistically significant compared with randomized data (*p*-values < 0.01).

The functional similarity between co-localized genes was assessed by comparing the GO term similarity with the spatial proximity values. Pearson correlation analysis did not detect any relationship between these variables. However, using the binning approach combined with the assessment of statistical significance (see Methods), we found that the mean GO term similarity is significantly (*p*-value < 0.01) correlated with spatial proximity in both species (Fig 5, S7 and S8 Figs).

### Contacts between HOX clusters

We were unable to identify high confidence contacts between any HOX clusters in human or mouse. Only for one pair of clusters (HOXA and HOXB in mouse) paired end reads were observed, but this could be caused by Hi-C biases as indicated by a high background probability (p-value 0.10). Homeobox (Hox) genes encode transcription factors involved in embryo development, and they are known to be expressed in a specific order in many species [19,20]. It is known that Hox clusters tend to co-localize when they are repressed [30]. We were unable to detect contacts between these clusters in human and mouse embryonic cells. However, we cannot rule out that the sequencing depth in the underlying experiment was too low to capture such interactions.

### Co-expression and transcription factor binding of genes in HGIN spatial clusters

**Fig 6:**
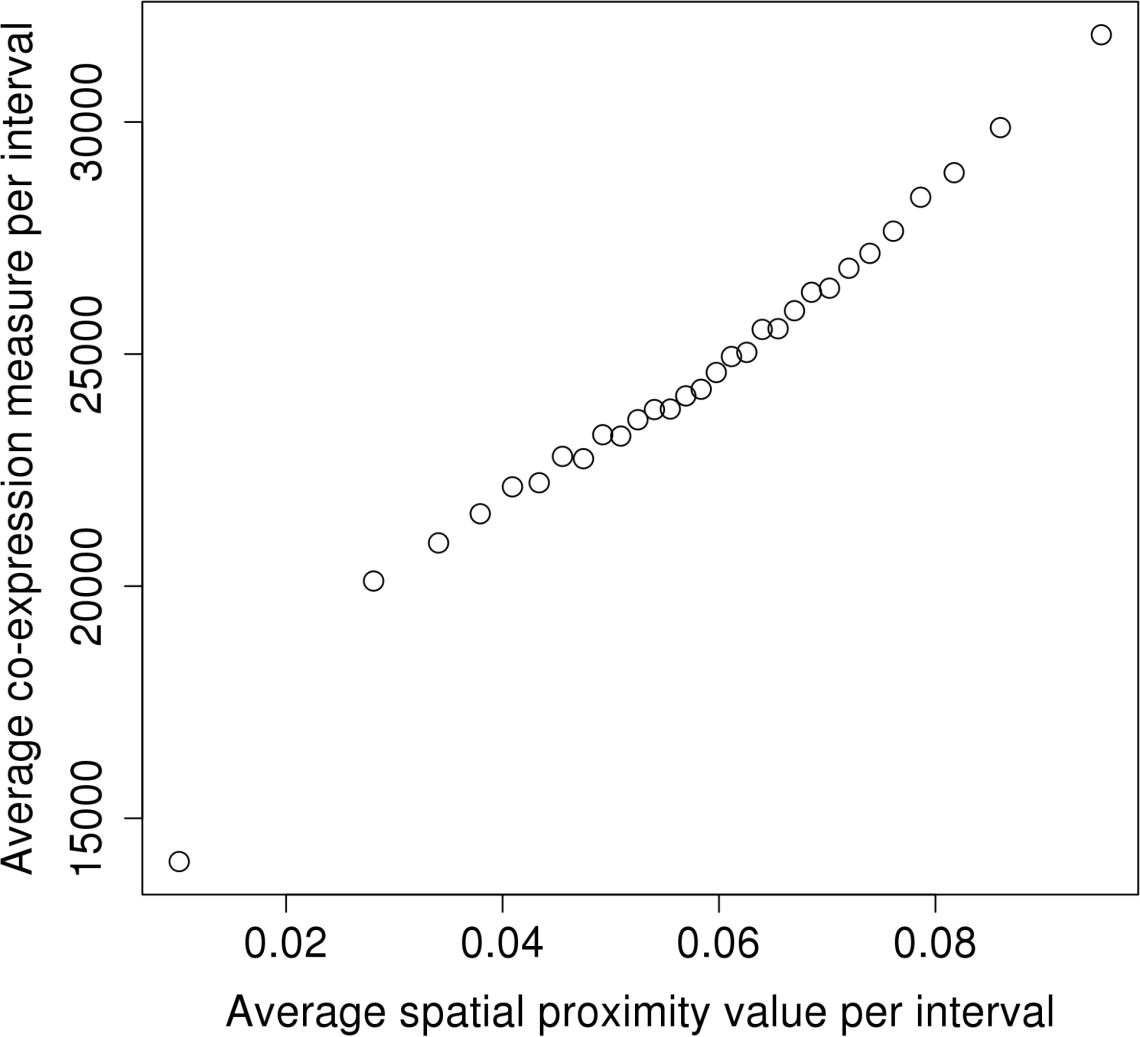
Correlation between the mean co-expression measure in the human genome and the mean spatial proximity value of 30 intervals. This is high and statistically significant compared with randomized binned data (*p*-value < 0.01). Co-expression was determined on the basis of the embryonic stem cell data set of Liu et al. (23) and the data were binned to reduce the effect of noise.

We also investigated the co-expression of genes located in close proximity in the HGIN, where we utilized gene expression profiles measured in human ESCs [24] to calculate a combined co-expression measure, as described by Khrameeva et al. [8]. Again, although the Pearson correlation coefficients were close to zero, using the binning procedure we found a strong and significant (S9 Fig) association between the mean spatial proximity values and the co-expression measure (Fig 6), which agreed with the results obtained by Khrameeva et al. [8] for fibroblasts.

We analyzed the preference for certain transcription factors in the spatially clustered genes, and we detected average CTCF- and RAD21-binding frequencies of 56% and 63% of the genes in all clusters, respectively (S7 Table, S10 Fig). Thus, compared with all transcription factors, which bind on average only 19.54% of the genes in a cluster, these two proteins appear to play special roles in inter-chromosomal contacts. We also identified a number of transcription factors (NANOG, BCL11A, POU5F1, EZH2, SUZ12, SP2, JUN, RFX5, FOSL1, RXRA, KDM5A, CHD1, and BRCA1) that had no or very few binding sites in most spatial clusters (S10 Fig).

### Evolutionary conservation of inter-chromosomal gene contacts

Among the 3,207 regions with conserved gene order in the human and mouse genomes, only 278 regions have overlaps in their spatial contacts, which involve only 1% of all genes. After randomly shuffling the orthologous regions, 234 synteny regions had overlaps with spatial clusters, thereby indicating that the observed overlaps are not biologically significant. Therefore, we conclude that the inter-chromosomal gene interactomes are not conserved in the human and mouse genomes.

Previous research has shown that intra-chromosomal contacts are largely conserved in the human and mouse genomes [6]. However, the inter-chromosomal landscape is more complex because of the high number of macrorearrangements in the genomes of the two species. Thus, the three-dimensional genome folds are expected to dramatically differ in both species.

## Discussion

In this study, we adapted the network framework for Hi-C data, which was initially applied to the yeast genome [9], to the human and mouse Hi-C data published by Dixon et al. [6]. This framework encompasses normalization of the data to remove noise and known biases as well as a probabilistic approach to detect high-confidence interactions. We constructed SINs and GINs for the inter-chromosomal contacts in both species and compared them at different confidence levels. To circumvent the low read coverage in inter-chromosomal mammalian Hi-C experiments, we binned data into 500 kb segments. As a consequence, the resolution of our analyses is relatively low and associations may appear weaker. In addition, the lower signal-to-noise ratio compared to contacts within chromosomes can obscure trends, and the results presented here should be considered with caution.

Networks are ubiquitously used in computational biology to understand complex data such as protein–protein–interaction networks or regulatory networks. For Hi-C data, the most commonly used visualization method is based on heat maps; however these can only illustrate pairwise interactions and this makes it difficult to identify the underlying metastructures directly. Using the network approach, we detected a strong non-random clustering behavior in both the human and mouse genomes. This clustering was previously observed in the yeast inter-chromosomal contact network [9], where it was considered to be caused by centromere co-localization. It is conceivable that some of the observed inter-chromosomal contacts are caused by chromosomal aberrations such as large-scale translocations or duplications, which cannot be detected using only Hi-C data. Further experimental validation using fluorescent in situ hybridization (FISH) might shed light on this matter.

### Human and mouse genomes show centromere clustering and a flexible Y chromosome

We detected co-localization of the centromeric regions of all chromosomes, with a highly flexible chromosome 11 region in mouse ESCs. This finding agrees with previous reports of centromere aggregation in several mouse cell types, which were discovered by FISH and visual interpretation as early as 1971 [27]. According to our data, all the centromere-adjacent regions form contacts with a single segment on chromosome 11. We suggest that this segment is centrally located in a loose cluster of centromeres, thereby allowing it to make contact with many of them, whereas the contacts between the centromeres of other chromosomes are apparently less dominant.

We also observed a higher contact density in the genome regions adjacent to centromeric regions in many human chromosomes (Fig 2). In differentiated human cell types, the centromeres tend to be located at the nuclear periphery, or at the nucleolus, whereas they are located more centrally in hESCs [26]. According to Wiblin et al. [26], centromeres appear in 34 clusters in this cell type, thereby implying that some smaller spatial clusters of multiple chromosome centromeres do exist. This agrees with our results, which show that there is a higher contact frequency for some pairs of chromosomes, e.g., 3 and 6, instead of high connectivity between all pairs of centromeres.

Interestingly, there is another high-degree region on chromosome Y in mouse, which forms almost 3,000 contacts with the rest of the genome. We suggest that the existence of these highly interacting segments in mouse is not caused by experimental bias. First, in Dixon et al.’s experiment, reads from two different locations in the genome, which are spatially close, were sequenced as paired end reads and mapped *uniquely* to the genome, and background probability calculation with *hicpipe* also considers fragment uniqueness. Second, reads that map to chromosome Y (2,500,000 bp to 3,000,000 bp), are paired with the reads from many other different locations, which makes it even less likely that this is a random result. Although both these segments contain a high percentage of repeats (segment on chromosome 11: 4.6 times higher than average; segment on chromosome Y: 2.8 times higher than average), this did not lead to an overestimation of the contacts because of the unique mapping procedure. While we cannot completely rule out the possibility that these findings are caused by low quality of the underlying genome build at pericentromeric or sub-telomeric regions, the strength of the observed effect in he mouse genome and the absence of a similar trend in the human genome provide an indication that these findings observations are in fact valid.

The short and gene-less genome area on chromosome Y could not form this many contacts simultaneously; thus, we assume that it is highly flexible and capable of forming contacts with many different loci in different cells. The Y chromosome exhibits remarkable structural differences compared with the X chromosome and autosomes; therefore, it is often excluded from analyses. Our results demonstrate that it also plays a unique role in the mouse interactome. The majority of the mouse Y chromosome (95%) consists of internally repetitive 515 kb units, which are repeated 150–200 times, whereas only 3 Mb originates from the ancestral autosome pair from which the X and Y chromosomes evolved [31]. Consequently, in Dixon et al.’s experiment, only these 3 Mb of the mouse Y chromosome could be uniquely mapped after sequencing, where the highly interactive segment lies closest to the repeat-rich long arm of the chromosome. In the human genome, we found that the similarly repeat-rich chromosome Y was also involved in a higher number of interactions than other longer chromosomes, although to a lesser extent. Because the female genome does not contain a Y chromosome, the observed high contact numbers are presumably not indispensable for the structure of the chromatin interactome. We suggest that the unmappable part of the chromosome Y, which contains a large portion of repeats and their adjacent regions, may be less embedded in the inter-chromosomal contact network; thus it may move around and form random contacts more freely than other chromosomes. Because these regions are very gene-poor, the functional contacts that need to be maintained are rare, which hypothetically allows for higher mobility.

The main structural feature of the human genome highlighted by our study is a higher incidence of contacts involving shorter chromosomes. In the nuclear architecture model described in Towbin et al. [32] and indicated in many studies of mammalian cell types [33,34], the chromosomes are organized into two zones, with transcriptionally inactive regions situated at the nuclear periphery and more active regions located closer to the center of the nucleus. In addition, the chromosomes tend to be localized in their own territories [4]. Although chromatin intermingling occurs, with chromatin loops penetrating other territories, the surface of a short chromosome territory is still larger in relation to its length than that of a long chromosome. Thus, this feature and the central localization could lead to the formation of a high number of contacts between short chromosomes because of architectural rather than functional reasons.

According to tethered conformation capture data, human chromosome territories can be assigned to two main spatial zones on the basis of distance-based clustering [28]. In agreement with the nuclear architecture model, the first of these groups is located in a central subnuclear region and it consists of chromosomes 1, 11, 14–17 and 19–22, which are relatively gene-rich. Our results confirm high interaction frequency between chromosomes 14–17 and 19–22, as well as between these chromosomes and others, which are logical consequences of their central positions. The remaining chromosomes preferentially reside at the nuclear periphery as part of the second group. Overall, our observations of more interactive short chromosomes agree with this model. For example, chromosome 19, which is only slightly shorter than chromosome 18 (chr19: 59.1 Mb, chr18: 78.1 Mb), but has a considerably higher gene number (chr19: 3,004, chr18: 1,209 genes) and thus density, also has 1.3 times the number of contacts (2,053 *vs*. 1,629). However, we could not distinguish between the groups for all chromosomes only on the basis of the normalized Hi-C data (e.g., chromosome 11 had similar interaction patterns to chromosome 12, although they belong to different groups).

### Human trans-interacting segments are enriched in active marks

The differences we detected between the human and mouse interactomes are not limited only to the overall network structure, because they also involve the genomic properties of trans-interacting segments. Active marks such as H3k4me3 are highly enriched in the auotosomes of the human genome, whereas the feature profiles of the mouse segments that form inter-chromosomal contacts are very similar to non-contact segments. It has already been shown in human that trans-interacting segments are enriched with active marks [3, 29], whereas no such correlation has been reported for mice. Our results indicate that this effect is not conserved between the species. However, it is possible that this observation is merely caused by differences in the differentiation stages of the used cells. For instance, the used mESCs could have been in a stagnant phase in contrast to active proliferation, causing a lack of contact formation between active regions.

In human, a high contact density is exhibited by the short gene-rich chromosomes. Because a high gene density naturally coincides with a higher number of active marks, this strong involvement of gene-rich chromosomes is presumably related to the increased numbers of active marks in the trans-interacting regions.

### Spatial proximity associates with co-expression in the human genome

To determine whether functional roles are related to the proximity of genes in the three-dimensional space, we performed correlation analysis using the average spatial proximity values and the average co-expression measure (described in S1 Methods). In general, chromosomes keep to their designated territories, and the co-localization of genes may occur because of the inherent random Brownian motions of chromosomes [35]. On the other hand, it is also possible that genes within spatial clusters are co-localized to facilitate gene regulation or co-expression. The expression of proximal genes may be controlled by the same set of regulatory elements, and the reuse of the already present transcription machinery could greatly enhance the speed and efficiency of expression for the co-localized genes. A previous study by Khrameeva et al. [8] based on Hi-C data from human fibroblasts detected a positive correlation between spatial proximity and co-expression values. By applying the method proposed by Khrameeva et al. to the hESC data obtained by Liu et al. [24], we were able to reproduce their results for this undifferentiated cell type, and we detected a significant association between co-expression and spatial proximity intervals in the human genome. While this association appears weak, it is possible that a higher-resolution analysis of data with better read coverage could find a stronger correlation.

### HOXB and HOXC clusters do not co-localize in human and mouse embryonic stem cells

We were not able to detect physical contacts between HOX clusters in human or mouse. Since Hox genes are known to be localized in compact clusters upon repression [30], this negative finding might be caused by the fact that these genes are indeed expressed in the stem cells considered here. However, we also have to take into account the possibility that low sequencing depth hides any Hox contacts. If for example only currently active genes are looped out of the spatial Hox cluster, we should have been able to observe some contacts due to the averaging of Hi-C data over millions of cells. As Hi-C data with higher resolution becomes available, further investigation of HOX clusters could provide more information on this structure.

### GO term similarity associates with spatial proximity in both species

Khrameeva et al. [8] reported correlations between the GO term similarity and spatial proximity in fibroblasts. We confirmed their results in human ESCs and also demonstrated that such correlations exist in mouse ESCs based on statistically significant correlations between the average spatial proximity values and GO term similarity (*p*-values < 0.01). We believe that the rather weak magnitude of this effect is caused by the low resolution of our analysis. Thus, although dramatic structural rearrangements occur during differentiation, stem cells and differentiated cells generally preserve the contacts between regions with functional similarity and similar expression profiles. These results agree with the concept of transcription factories, where genes with similar functions come close together in space to facilitate their co-expression. Notably, this effect is present in both the human and mouse genomes despite apparent differences in their genome structures.

### CTCF and RAD21 bind most genes in HGIN spatial clusters

In spatial clusters, on average, 56% and 63% of the co-localized human genes have binding sites for CTCF and RAD21, respectively. Both these transcription factors are known to be involved in spatial chromatin organization, where RAD21 is a subunit of the cohesin complex and plays a role in sister chromatid cohesion [36–40]. Chromatin conformation studies have also shown that cohesin can form long-range interactions between its binding sites, thereby establishing and maintaining long-distance or even inter-chromosomal interactions [41–43]. Because most genes in small isolated spatial clusters have a RAD21-binding site, cohesin may be the protein responsible for the initiation of these contacts. Similarly, the highly conserved CTCF is required for long-range interactions [44]. Its putative functions include insulator activities, imprinting, promoter activation and repression, and the facilitation of long-distance contacts [40,45,46]. It has been shown that cohesin can stabilize CTCF binding [40,44]. However, CTCF is also thought to function as a recruiting factor for cohesin [40,44,47]. Thus, the abundance of CTCF- and RAD21-binding sites in the co-localized gene clusters is not surprising. The average number of binding sites for CTCF and RAD21 in the segments with inter-chromosomal contacts is higher than that in other segments, thereby confirming the important role of these transcription factors in trans-interactions with the rest of the genome.

### Inter-chromosomal contacts are not conserved between human and mouse

Previously, it was shown that the domain structure of intra-chromosomal contacts is conserved in the human and mouse genomes. However, using the same data, we found that the inter-chromosomal contacts are not conserved. Thus, it is apparent that the multiple rearrangements of large genomic blocks (synteny regions) after humans and mice diverged from their common ancestor disrupted ancient chromosome territories. We found that the conservation of contacts in regions with conserved gene orders was not higher than that expected by chance.

The co-localization of many centromeric regions (mouse) and short chromosomes (human) could be observed at the nuclear scale, thereby suggesting a general difference in the organization of trans-interactions. The individual inter-chromosomal interactions are not conserved and have different genomic properties in the human and mouse genomes, indicating that these interactions have little functional purpose.

However, at the functional level, we found that the properties of the human and mouse interactomes are conserved to some extent. Both species have strong correlations between GO term similarity and spatial proximity, which indicates the existence of equivalent transcription factories. In addition, they shared structural characteristics such as a (partially) flexible Y chromosome and a tendency for shorter chromosomes to form more contacts.

Because the spatial structure of the genome is reorganized during differentiation, including re-locations of entire chromosomes, similar analysis using a differentiated cell type may lead to different results. Ultimately, a network-based interpretation of the complete human and mouse chromatin interactomes at different stages of differentiation would provide a more complete picture of chromatin organization.

## Supporting Information

S1 Methods Supplementary information on the methods used in this publication. (PDF)

S1 Table Description of features used for enrichment analysis of trans-interacting segments. (PDF)

S2 Table Size of segment interaction networks at different q-value cutoffs in human and mouse. 1^st^ component is the first and largest connected component. In both species almost all segments are either part of the first component or not connected at all.
(PDF)

S3 Table Sizes of random gene interaction networks in human and mouse for 10 different runs. We chose run 9 in human and run 3in mouse and their corresponding segment interaction networks as a representative random network for detailed comparisons, since these have sizes closest to the average of all runs.
(PDF)

S4 Table Overlap of genomic features with trans-interacting segments and other segments in human and mouse. (PDF)

S5 Table Average number of transcription factor binding sites for 55 transcription factors in trans-interacting and not trans-interacting segments. CTCF and RAD21 show the highest increase of binding sites in segments with inter-chromosomal contacts.
(PDF)

S6 Table Basic network statistics for different q-value cutoffs and corresponding gene interaction networks in both species. Number of bins describes the number of bins with genes with at least one connection to other genes present in the network. Genes per bin are calculated for these present segments only. Number of singletons refers to genes without any contacts. Degree of network connectivity drastically decreases with decreasing q-value threshold.
(PDF)

S7 Table Average percentage of genes in a spatial cluster with TFBS for the listed 55 transcription factors. CTCF and RAD21 bind around two thirds of genes in spatial clusters on average. The mean of all 55 transcription factors is 19.54%.
(PDF)

S1 Fig Illustration of conservation analysis. For each syntenic region A, the genes in contact with genes in A were determined for both organisms, rendering sets *H* and *M*. For comparison of these sets, genes in *H* were translated into their mouse orthologs (set *H_M_*) and overlap to *M* was determined.
(PNG)

S2 Fig Circos visualization of gene contacts in the random mouse segment interaction network (RMSIN). Banded ideograms represent the chromosomes of the mouse genome, black lines indicate a contact in the RMSIN. Contacts are distributed very regularly.
(PNG)

S3 Fig Circos visualization of the random human segment interaction network (RHSIN). Colored ideograms represent the chromosomes of the human genome, black lines indicate a contact in the RHSIN. Contacts are distributed regularly.
(PNG)

S4 Fig Relationship of chromosome length and average degree in the MSIN after exclusion of outliers chromosome 11 and chromosome Y, log-log scale. Pearson correlation coefficient -0.87.
(PNG)

S5 Fig Distribution of degree and shortest path length for the human segment interaction network. Degree distribution is shown as a log-log plot. Low degrees are very common, while higher degrees are less frequent. The red line is the fitted power law function with slope -1.896, the fit’s correlation is 0.983. Shortest path length is normally distributed and centers around a medium path length of 4.5, which is mainly caused by the existence of hubs and the power-law degree distribution.
(PNG)

S6 Fig Distribution of degree and shortest path length for the mouse segment interaction network. Degree distribution is shown as a log-log plot. Due to its high connectivity, (maximum) shortest path length is short and very high degrees are observable. The red line shows the fitted power law function with slope -0.752, the fit’s correlation is 0.914, though the two extreme hubs (at degrees of 1,000 and 4,000) disturb the fit.
(PNG)

S7 Fig Distribution of correlation coefficients between spatial proximity value and GO term similarity in human for 1000 randomized data sets and observed data (red line). According to cumulative distribution function (CDR) observed result is significant (p-value = 0).
(PNG)

S8 Fig Distribution of correlation coefficients between spatial proximity value and GO term similarity in mouse for 1000 randomized data sets and observed data (red line). According to CDR observed result is significant (p-value = 0).
(PNG)

S9 Fig Distribution of correlation coefficients between spatial proximity value and co-expression measure in human for 1000 randomized data sets and observed data (red line). According to CDR observed result is significant (p-value = 0).
(PNG)

S10 Fig Heatmap showing the percentage of genes per inter-chromosomal spatial cluster that overlap with any of 55 transcription factors’ binding sites. Blue color indicates no or little overlap between the genes in the cluster and the TFBS, red color indicates high overlap. There is a large section of transcription factors which rarely are involved in the genes of spatial clusters (NANOG to BRCA1), but also a set of TF that binds to these genes more often (USF1 to TAF1). CTCF, which is known to play a role in the structural organization of the genome, binds many of the genes in the clusters.
(PNG)
