# Supplementary material for: Inter-Chromosomal Contact Networks Provide Insights into Mammalian Chromatin Organization
Source: PLoS One. 2015 May 11;10(5):e0126125. doi: 10.1371/journal.pone.0126125 (PMC4427453; doi:10.1371/journal.pone.0126125)
Supplement: S1 Methods — (PDF) [file pone.0126125.s001.pdf]

Inter-chromosomal gene contact networks provide insights into mammalian chromatin organization

Supplementary Methods

Normalization

As proposed by Kruse et al (2013) and Dixon et al (2012), we used Yaffe and Tanay's method (2011) to exclude all known Hi-C biases, including correction of read mappability, elimination of nonspecific ligation products and length and GC content biases. We used the published method *hicpipe*, version 0.93, and experimental Hi-C data is taken in the form of the Hi-C summary file. HindIII cutting sites were found for human and mouse by use of R bioconductor (Gentleman et al. 2004) Biostrings for scanning of the complete genome sequences for the recognition sequence AAGCTT. Mappability score for fragment ends was calculated by division of the genome sequences into artificial 50bp reads, starting every 10bp, and mapping back to the genome with BWA (Li and Durbin, 2009) using default parameters.

Filtering

To discard unspecific contacts we used a q-value based approach as suggested by Kruse et al., but made adaptations to fit the lower read coverage and more complex genome structure of mammalian species. Instead of calculating the p-value of each fragment pair contact, we binned fragments over 500 kb to capture enough information given the low read coverage of 2.46 and 2.66 reads per fragment pair in human and mouse, respectively. Filtering was performed separately for each pair of chromosomes. The P-Value is calculated as follows:

$$P(bin_{a,}bin_b) = \sum_{i=k}^{n} \binom{n}{k} m_{norm}^{i} (1 - m_{norm})^{n-1}$$

Where $bin_{a,}bin_b$ are two 500kbp bins, m_{norm} is the average interaction probability of all pairs of fragments $frag_{a,}$ from $bin_{a,}$ and $frag_b$ from bin_b, k is the observed number of reads for $bin_{a,}bin_b$, and n is the total number of observed reads for the chromosomes $chr_{a,}chr_b$ from which $bin_{a,}bin_b$ stem.

To correct for multiple hypothesis errors, FDR is performed on the p-Values using the method by Benjamini and Hochberg (Benjamini and Hochberg 1995) that is integrated into R.

Use of *n* as the total number of observed interactions on the current pair of chromosomes could introduce a bias towards smaller chromosomes, which can harbor only a small *n* compared to longer chromosomes. We introduced a second normalization step to avoid this behavior. On each pair of chromosomes $chr_{a,}chr_b$ all q-values for interactions happening between them are normalized as follows:

$$q_{norm} = \frac{q}{l_{factor}} length(chr_a) * length(chr_b)$$

Where l_{factor} is the product of the length of the longest and second longest chromosome.

We created networks for a number of different q_{norm} cutoffs (see table S2) and decided to use the strict cutoffs of $1e-3$ and $1e-6$ for human and mouse, respectively, since for this threshold the human and mouse gene interaction network contain a similar percentage of genes without connections.

Randomization

Randomization of the gene interaction networks was performed according to Kruse et al. As a basis, we distributed all X segments of the human and X segments of the mouse genome randomly in a unicube. We inserted edges between the Y pairs of segments that were in closest proximity according to the Euclidean distance, where Y is the number of interactions from the original network. The random segment interaction networks of both species were then permuted on the level of edges and their transitivity raised to create a randomized network with similar properties as the originals with respect to clustering behavior and connectivity. For edge permutation, two random edges (u, v) and (s, t) are deleted and two new edges (u, t) and (s, v) are inserted into the network if

1. $u \neq t \wedge s \neq v$
2. (u, t) and (s, v) do not already exist
3. u and t, s and v are not from the same chromosome

As suggested by Kruse et al, the rewiring step is performed $10 \times |E|$ times. Raise of transitivity is implemented in a simple fashion, using the transitivity definition by Soffer and Vasquez (Bansal et al. 2009):

$$\tilde{T} = \frac{\sum_i \delta(i)}{\sum_i \omega(i)}$$

where i is a node in the network, $\delta(i)$ is the observed number of triangles in the neighborhood of i, i.e. the number of connected neighbors of i, and $\omega(i)$ is the maximum possible number of triangles in the neighborhood of i.

To prevent stagnation, we introduced a threshold of $10{,}000 \times |E|$ for maximum number of tries to raise the transitivity. In the case of human this was reached after a transitivity of 0.0038, comparing to the original network's transitivity of 0.0084. In mouse, transitivity was raised completely before the threshold was reached.

We performed randomization 10 times independently, and chose the run for which basic network properties most resembled the average of these 10 runs.

The randomized segment interaction network is transformed into a gene interaction network by assigning n random genes to each segment s, with n being the number of genes actually lying in s. We created 10 random gene interaction networks per species and continued to compare our results to two sample networks (run 9 in human, run 3 in mouse) which were closest in size to the average (table S3).

Calculation of spatial proximity values

Spatial proximity values are first defined in Lieberman-Aiden et al. (2009) and are based on the normalized Hi-C data. We applied a slightly modified version to the data from Dixon et al. to account for the background interaction probability calculated by *hicpipe*.

Spatial proximity values were calculated for the matrix of all-vs-all 500kb segment interactions, comprising the whole genomes of human and mouse, respectively. The observed matrix O derives directly from the data, containing the number of observed reads for each segment pair's interaction. For each such interaction the expected value was calculated by *hicpipe*, creating the expected matrix *E*.

The normalized interaction score for each cell (i,j) is calculated as suggested by Yaffe and Tanay (2011) using the formula

$$N[i,j] = \frac{O[i,j]}{E[i,j] * N[i] * N[j]}$$

where

$$N[i] = \frac{O[i]}{E[i]}$$

$$O[i] = \sum_j O[i,j]$$

$$E[i] = \sum_j E[i,j]$$

Matrix smoothing was not applied. Instead, we applied a natural logarithm transformation to the normalized matrices to increase the signal outside of the main diagonal region. The spatial proximity value was then calculated genome-wide for each cell $c_{i,j}$ of this ln-transformed normalized matrix as the Pearson correlation coefficient of rows i and j, which are identical to columns i and j due to symmetry of the matrix. For correlation analysis between co-expression/GO term similarity and spatial proximity, only inter-chromosomal contact pairs were considered.

Correlation between physical co-localization and co-expression of genes

Liu et al (2006) conducted a genome-wide expression profiling of human embryonic stem cells, resulting in expression values for over 20,000 genes in 43 hESC samples and allowing us to compare co-localization and co-expression of genes in Dixon et al.'s data with comparable cell types. Applying Khrameeva et al's method (2012), we transformed the data into a co-expression measure using the following method:

$$E(i,j) = \sum_{k=i}^{n} \left(\frac{W_{ki}}{N_i} + \frac{W_{kj}}{N_j} \right) * R_k$$

Where i and j are two genes from two interacting segments, W_{ki} and W_{kj} are the portion of the respective 500 kb segment that overlaps with gene i and j, respectively, N_i and N_j are the number of genes in the

corresponding segments and R_k is the pearson correlation coefficient of the two genes' expression profiles.

Transcription factor binding sites

To further investigate the relationship between co-localization and expression, we downloaded transcription factor binding site tracks from ENCODE/HAIB (March 2012) for CTCF, NANOG and POLR2A for hESC samples. We calculated the overlap of genes and TFBS peaks for each transcription factor.

Supplementary References

Bansal S, Khandelwal S and Meyers LA. 2009. Exploring biological network structure with clustered random networks. *BMC Bioinformatics* **10**: doi:10.1186/1471-2105-10-405.

Benjamini Y and Hochberg Y. 1995. Controlling the False Discovery Rate: a Practical and Powerful Approach to Multiple Testing. *J. R. Statist. Soc.* **57**(1): 289-300.

Dixon JR, Selvaraj S, Yue F, Kim A, Li Y, Shen Y, Hu M, Liu JS and Ren B. 2012. Topological domains in mammalian genomes identified by analysis of chromatin interactions. *Nature* **485**(7398): 376-380.

Gentleman RC, Carey VJ, Bates DM, Bolstad B, Dettling M, Dudoit S, Ellis B, Gautier L, Ge Y, Gentry J, et al. 2004. Bioconductor: open software development for computational biology and bioinformatics.
Genome Biol. **5(10)**: R80.

Khrameeva EE, Mironov AA, Fedonin GG, Khaitovich P, Gelfand MS. 2012. Spatial proximity and similarity of the epigenetic state of genome domains. *PLoS ONE* **7**(4): e33947, doi:10.1371/journal.pone.0033947.

Kruse K, Sewitz S and Babu MM. 2013. A complex network framework for unbiased statistical analyses of DNA-DNA contact maps. *Nucleic acids research* **41**: 701-710.

Li H and Durbin R. 2009 Fast and accurate short read alignment with Burrows-Wheeler Transform. *Bioinformatics* **25**:1754-60.

Lieberman-Aiden E, van Berkum NL, Williams L, Imakaev M, Ragoczy T, Telling A, Amit I, Lajoie BR, Sabo PJ, Dorschner MO, et al. 2009. Comprehensive mapping of long-range interactions reveals folding principles of the human genome. *Science* **326**: 289-293.

Liu Y, Shin S, Zeng X, Zhan M, Gonzalez R, Mueller F, Schwartz CM, Xue H, Li H, Baker SC, et al. 2006. Genome wide profiling of human embryonic stem cells (hESCs), their derivatives and embryonal carcinoma cells to develop base profiles of U.S. Federal government approved hESC lines. *BMC Dev Biol.* **6**(20).doi: 10.1186/1471-213X-6-20.

Yaffe E and Tanay A. 2011. Probabilistic modeling of Hi-C contact maps eliminates systematic biases to characterize global chromosomal architecture. *Nature Genetics* **43**: 1059-1065.
