# Supplementary material for: Inter-Chromosomal Contact Networks Provide Insights into Mammalian Chromatin Organization
Source: PLoS One. 2015 May 11;10(5):e0126125. doi: 10.1371/journal.pone.0126125 (PMC4427453; doi:10.1371/journal.pone.0126125)
Supplement: S1 Table — (PDF) [file pone.0126125.s002.pdf]

S1 Table. Description of features used for enrichment analysis of trans-interacting segments.

FEATURE	DESCRIPTION	CELL TYPE	SOURCE
		H. sapiens	
**H3K4ME3 PEAKS**	Active mark	hESC	ENCODE, Broad Institute, hg18
**H3K4ME1 PEAKS**	Active mark	hESC	ENCODE, Broad Institute, hg18
**H3K27AC PEAKS**	Active mark	GM12878	ENCODE, Broad Institute, hg18
**H3K9AC PEAKS**	Active mark	hESC	ENCODE, Broad Institute, hg18
**H3K36ME3 PEAKS**	Mark of transcribed regions	hESC	ENCODE, Broad Institute, hg18
**LADS**	Lamina associated domains	Fibroblasts	NKI, Peric-Hupkes et al, hg18
**DNASE I SITES**	Accessible sites	Collection of cell types	ENCODE DNase clusters, hg18
**LINE REPEATS**		-	RepeatMasker, hg18
**LTR REPEATS**		-	RepeatMasker, hg18
**NUCLEOSOME OCCUPANCY**		-	Predicted, UW, hg18
**OPEN CHROMATIN**		GM12878	ENCODE, Duke-UNC-UT, hg18
**RTD**	Replication timing domains	Lymphoblasts	ReplicationDomain DB, hg18
**SINE REPEATS**		-	RepeatMasker, hg18
		M. musculus	
**H3K4ME3 PEAKS**	Active mark	ES-E14	ENCODE/LICR, mm9
**H3K4ME1 PEAKS**	Active mark	ES-E14	ENCODE/LICR, mm9
**H3K27AC PEAKS**	Active mark	ES-E14	ENCODE/LICR, mm9
**H3K9AC PEAKS**	Active mark	ES-E14	ENCODE/LICR, mm9
**H3K36ME3 PEAKS**	Mark of transcribed regions	ES-E14	ENCODE/LICR, mm9
**LADS**	Lamina associated domains	mESC	NKI, Peric-Hupkes et al, mm9
**DNASE I SITES**	Accessible sites	ES-E14	ENCODE/University of Washington, mm9
**LINE REPEATS**		-	RepeatMasker, mm9
**LTR REPEATS**		-	RepeatMasker, mm9
**OPEN CHROMATIN**		-	ENCODE/Duke/UNC/UT, mm9
**RTD**	Replication timing domains	mESC	ReplicationDomain DB, mm9
**SINE REPEATS**		-	RepeatMasker, mm9
