# Supplementary material for: Inter-Chromosomal Contact Networks Provide Insights into Mammalian Chromatin Organization
Source: PLoS One. 2015 May 11;10(5):e0126125. doi: 10.1371/journal.pone.0126125 (PMC4427453; doi:10.1371/journal.pone.0126125)
Supplement: S2 Table — 1st component is the first and largest connected component. In both species almost all segments are either part of the first component or not connected at all. (PDF) [file pone.0126125.s003.pdf]

Table S2. Size of ***segment interaction networks*** *at different q-value cutoffs in human and mouse. 1st component is the first and largest connected component. In both species almost all segments are either part of the first component or not connected at all.*

CUTOFF	#NODES	#CONNECTED NODES	#EDGES	1ST COMPONENT #NODES	#EDGES	2ND COMP. #NODES
	H. sapiens					
**0.05**	5,732	5,254 (91.66%)	31,401	5,250	31,399 (99.99%)	2
**1E-2**		4,128 (72.02%)	13,674	4,077	13,647 (99.81%)	3
**1E-3**		**2,500 (43.62%)**	**4,520**	**2,349**	**4,435 (98.12%)**	**5**
**1E-4**		1,342 (23.41%)	1,736	1,126	1,611 (92.80%)	7
**1E-5**		858 (14.97%)	989	692	889 (89.89%)	7
**1E-6**		483 (8.43%)	500	294	378 (38.22%)	14
**1E-8**		233 (4.07%)	238	90	142 (59.66%)	13
	M. musculus					
**0.05**	5,093	4,389 (86.18%)	6,731	4,383	6,729 (99.98%)	2
**1E-2**		4,363 (85.67%)	6,483	4,357	6,481 (99.97%)	2
**1E-3**		4,011 (78.76%)	5,589	4,003	5,586 (99.95%)	2
**1E-4**		3,820 (75.01%)	5,133	3,807	5,127 (99.88%)	3
**1E-5**		3,772 (74.06%)	4,978			
**1E-6**		**3,452 (67.78%)**	**4,483**	**3,428**	**4,470 (99.71%)**	**3**
**1E-8**		3,133 (61.52%)	3,953	3,105	3,938 (99.65%)	3
