# Supplementary material for: Inter-Chromosomal Contact Networks Provide Insights into Mammalian Chromatin Organization
Source: PLoS One. 2015 May 11;10(5):e0126125. doi: 10.1371/journal.pone.0126125 (PMC4427453; doi:10.1371/journal.pone.0126125)
Supplement: S3 Table — We chose run 9 in human and run 3in mouse and their corresponding segment interaction networks as a representative random network for detailed comparisons, since these have sizes closest to the average of all runs. (PDF) [file pone.0126125.s004.pdf]

S3 Table. Sizes of random gene interaction networks in human and mouse for 10 different runs. We chose run 9 in human and run 3in mouse and their corresponding segment interaction networks as a representative random network for detailed comparisons, since these have sizes closest to the average of all runs.

RUN	#NODES	#EDGES	#SINGLETONS	#CLUSTER	#NODES (1ST COMP.)	#EDGES (1ST COMP.)
			**RHGIN**			
**1**	20,229	51,945	6,368	458	5,379	23,229
**2**		54,050	6,190	435	5,743	25,926
**3**		50,592	6,464	448	5,116	23,229
**4**		57,092	6,394	403	6,861	32,608
**5**		59,267	5,996	450	7,011	36,055
**6**		54,078	6,108	496	4,911	22,782
**7**		55,071	6,483	436	6,528	30,925
**8**		54,656	6,080	489	5,949	26,075
**9**		**55,470**	**6,245**	**415**	**7,457**	**34,327**
**10**		57,080	6,258	449	6,060	29,807
**Ø**		54,930.1	6,258.6	447.9	6,101.5	28,496.3
			**RMGIN**			
**1**	22,341	77,795	6,479	358	9,500	51,415
**2**		81,571	6,449	283	10,865	60,802
**3**		**79,727**	**6,217**	**302**	**11,212**	**63,390**
4		79,185	6,341	308	10,577	58,687
**5**		79,171	5,857	334	11,060	61,148
**6**		84,765	6,064	290	11,173	65,266
7		81,845	6,363	309	10,536	62,055
**8**		79,853	6,440	288	11,031	62,475
**9**		81,057	6,501	309	10,430	59,991
**10**		79,567	6,369	318	9,869	56,887
**Ø**		80,453.6	3,608.0	309.9	10,625.3	60,211.6
