# Supplementary material for: Inter-Chromosomal Contact Networks Provide Insights into Mammalian Chromatin Organization
Source: PLoS One. 2015 May 11;10(5):e0126125. doi: 10.1371/journal.pone.0126125 (PMC4427453; doi:10.1371/journal.pone.0126125)
Supplement: S4 Table — (PDF) [file pone.0126125.s005.pdf]

S4 Table. Overlap of genomic features with trans-interacting segments and other segments in human and mouse.

	TOTAL OVERLAP WITH TRANS-INTERACTING SEGMENTS (%)	***TOTAL OVERLAP WITH NOT TRANS-INTERACTING SEGMENTS (%)***
	M. musculus	
**H3K4ME3**	1.77	1.76
**H3K4ME1**	5.45	5.89
**H3K27AC**	1.91	1.90
**H3K9AC**	2.01	2.02
**H3K36ME3**	3.99	4.14
**LADS**	14.27	12.80
**DNASE I SITES**	0.86	0.89
**LINE REPEATS**	15.82	15.64
**LTR REPEATS**	8.84	8.60
**OPEN CHROMATIN**	1.09	1.14
**RTD**	0.65	0.64
**SINE REPEATS**	6.34	6.71
	H. sapiens	
**H3K4ME3**	15.60	12.64
**H3K4ME1**	12.01	8.75
**H3K27AC**	3.39	2.66
**H3K9AC**	4.68	3.21
**H3K36ME3**	13.34	10.75
**LADS**	18.72	15.85
**DNASE I SITES**	9.31	8.00
**LINE REPEATS**	19.25	21.40
**LTR REPEATS**	7.98	8.74
**OPEN CHROMATIN**	2.86	2.18
**RTD**	4.55	4.51
**SINE REPEATS**	15.41	12.49
