# Supplementary material for: Inter-Chromosomal Contact Networks Provide Insights into Mammalian Chromatin Organization
Source: PLoS One. 2015 May 11;10(5):e0126125. doi: 10.1371/journal.pone.0126125 (PMC4427453; doi:10.1371/journal.pone.0126125)
Supplement: S5 Table — CTCF and RAD21 show the highest increase of binding sites in segments with inter-chromosomal contacts. (PDF) [file pone.0126125.s006.pdf]

S5 Table. Average number of transcription factor binding sites for 55 transcription factors in trans-interacting and not trans-interacting segments. CTCF and RAD21 show the highest increase of binding sites in segments with inter-chromosomal contacts.

	**TRANS-INTERACTING SEGMENTS**	**NOT TRANS-INTERACTING SEGMENTS**	**ENRICHMENT/DEPLETION**
**ATF2**	1.19	0.93	+0.26
**ATF3**	0.86	0.82	+0.04
**BACH1**	2.25	1.79	+0.46
**BCL11A**	0.57	0.34	+0.23
**BRCA1**	0.37	0.34	+0.03
**CEBPB**	3.33	2.24	+1.09
**CHD1**	0.44	0.34	+0.10
**CHD2**	1.24	1.16	+0.08
**CTBP2**	1.41	1.10	+0.31
**CTCF**	10.91	8.28	**+2.63**
**EGR1**	1.65	1.42	+0.23
**EP300**	1.79	1.38	+0.41
**EZH2**	1.29	0.96	+0.33
**FOSL1**	0.21	0.19	+0.02
**GABPA**	0.96	1.00	-0.04
**GTF2F1**	0.65	0.60	+0.05
**HDAC2**	1.14	0.86	+0.28
**JUN**	0.43	1.35	-0.92
**JUND**	1.63	0.33	+1.30
**KDM5A**	0.28	0.28	-
**MAFK**	2.50	1.60	+0.90
**MAX**	2.14	1.78	+0.36
**MXI1**	1.12	1.10	+0.02
**MYC**	0.85	0.75	+0.10
**NANOG**	1.17	0.79	+0.38
**NRF1**	0.81	0.77	+0.04
**POLR2A**	3.74	3.38	+0.36
**POU5F1**	0.86	0.57	+0.29
**RAD21**	15.64	11.27	**+4.37**
**RBBP5**	3.17	2.54	+0.63
**REST**	2.65	2.05	+0.60
**RFX5**	0.30	0.29	+0.01
**RXRA**	0.26	0.20	+0.06
**SIN3A**	3.81	3.64	+0.17
**SIN3AK20**	1.60	1.54	+0.06
**SIX5**	0.58	0.61	+0.03
**SP1**	2.78	2.52	+0.26
**SP2**	0.41	0.44	-0.03
**SP4**	1.03	0.98	+0.05
**SRF**	1.00	0.80	+0.20
**SUY12**	0.91	0.75	+0.26
**TAF1**	3.83	3.38	+0.45
**TAF7**	1.95	1.73	+0.22
**TBP**	3.20	2.84	+0.36
**TCF12**	1.55	1.22	+0.33
**TEAD4**	4.18	2.90	+1.28
**USF1**	5.16	4.06	+1.10
**USF2**	1.31	1.13	+0.17
**YY1**	3.53	2.93	+0.60
**YNF143**	5.76	5.02	+0.74
