# Supplementary material for: Inter-Chromosomal Contact Networks Provide Insights into Mammalian Chromatin Organization
Source: PLoS One. 2015 May 11;10(5):e0126125. doi: 10.1371/journal.pone.0126125 (PMC4427453; doi:10.1371/journal.pone.0126125)
Supplement: S6 Table — Number of bins describes the number of bins with genes with at least one connection to other genes present in the network. Genes per bin are calculated for these present segments only. Number of singletons refers to genes without any contacts. Degree of network connectivity drastically decreases with decreasing q-value threshold. (PDF) [file pone.0126125.s007.pdf]

S6 Table. Basic network statistics for different q-value cutoffs and corresponding ***gene interaction networks*** *in both species. Number of bins describes the number of bins with genes with at least one connection to other genes present in the network. Genes per bin are calculated for these present segments only. Number of singletons refers to genes without any contacts. Degree of network connectivity drastically decreases with decreasing q-value threshold.*

	H. SAPIENS					M. MUSCULUS				
**CUTOFF**	#Bins	#Genes per Bin	#Edges	#Nodes	#Singletons	#Bins	#Genes per Bin	#Edges	#Nodes	#Singletons
**0.05**	3,964	4.70	752,562	20,229	1,584 (7.83%)	1,557	5.70	123,392	22,341	13,468 (60.28%)
**1E-2**	2,996	4.94	299,487		5,420 (26.79%)	1,446	5.56	99,811		14,297 (63.99%)
**1E-3**	1,869	5.45	110,433		9,946 (49.17%)	1,250	5.70	77,562		14,983 (67.07%)
**1E-4**	**1,005**	**5.95**	**41,483**		**14,151 (69.95%)**	**1,134**	**5.30**	**66,331**		**15,798 (70.71%)**
**1E-5**	598	6.17	20,990		16,437 (81.25%)	1,046	5.33	58,633		16,234 (72.66%)
**1E-6**	335	6.20	9,530		18,050 (89.23%)	970	5.30	53,776		16,670 (74.62%)
**1E-8**	166	5.58	3,831		19,201 (94.92%)	861	5.22	45,932		17,310 (77.48%)
**1E-10**	77	4.70	1,162		19,766 (97.71%)	767	5.11	39,914		17,890 (80.08%)
**1E-12**	69	4.68	1,096		19,805 (97.90%)	698	5.04	34,097		18,290 (81.87%)
