# Supplementary material for: Inter-Chromosomal Contact Networks Provide Insights into Mammalian Chromatin Organization
Source: PLoS One. 2015 May 11;10(5):e0126125. doi: 10.1371/journal.pone.0126125 (PMC4427453; doi:10.1371/journal.pone.0126125)
Supplement: S7 Table — CTCF and RAD21 bind around two thirds of genes in spatial clusters on average. The mean of all 55 transcription factors is 19.54%. (PDF) [file pone.0126125.s008.pdf]

S7 Table. Average percentage of genes in a spatial cluster with TFBS for the listed 55 transcription factors. CTCF and RAD21 bind around two thirds of genes in spatial clusters on average. The mean of all 55 transcription factors is 19.54%.

**ATF2**	**ATF3**	**BACH1**	**BCL11A**	**BRCA1**	**CEBPB**	**CHD1**
8.10%	8.60%	21.54%	2.97%	2.31%	23.13%	
**CHD2**	**CTBP2**	**CTCF**	**EGR1**	**EP300**	**EZH2**	**FOSL1**
16.62%	9.05%	55.73%	21.01%	16.15%	3.16%	0.70%
**GABPA**	**GTF2F1**	**HDAC2**	**JUN**	**JUND**	**KDM5A**	**MAFK**
11.49%	7.32%	9.18%	2.51%	17.00%	2.41%	15.04%
**MAX**	**MXI1**	**MYC**	**NANOG**	**NRF1**	**POLR2A**	**POU5F1**
21.84%	15.53%	8.65%	8.16%	9.77%	50.56%	5.65%
**RAD21**	**RBBP5**	**REST**	**RFX5**	**RXRA**	**SIN3A**	**SIN3AK20**
63.33%	45.27%	19.98%	2.17%	1.62%	53.05%	23.16%
**SIX5**	**SP1**	**SP2**	**SP4**	**SRF**	**SUY12**	**TAF1**
5.94%	34.67%	3.39%	14.52%	7.17%	3.12%	57.41%
**TAF7**	**TBP**	**TCF12**	**TEAD4**	**USF1**	**USF2**	**YY1**
30.94%	51.22%	10.92%	27.17%	41.98%	11.98%	40.59%
**YNF143**						
50.80%						
