# Supplementary figures and images for: Inter-Chromosomal Contact Networks Provide Insights into Mammalian Chromatin Organization
Source: PLoS One. 2015 May 11;10(5):e0126125. doi: 10.1371/journal.pone.0126125 (PMC4427453; doi:10.1371/journal.pone.0126125)

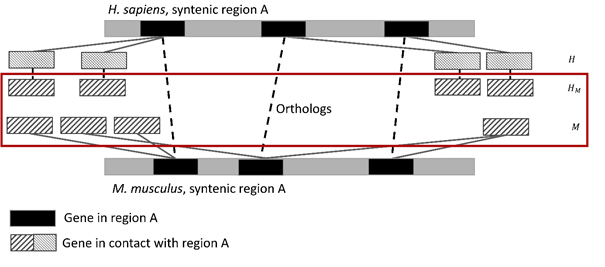

Supplement: S1 Fig — For each syntenic region A, the genes in contact with genes in A were determined for both organisms, rendering sets H and M. For comparison of these sets, genes in H were translated into their mouse orthologs (set HM) and overlap to M was determined. (PNG) [file pone.0126125.s009.png]

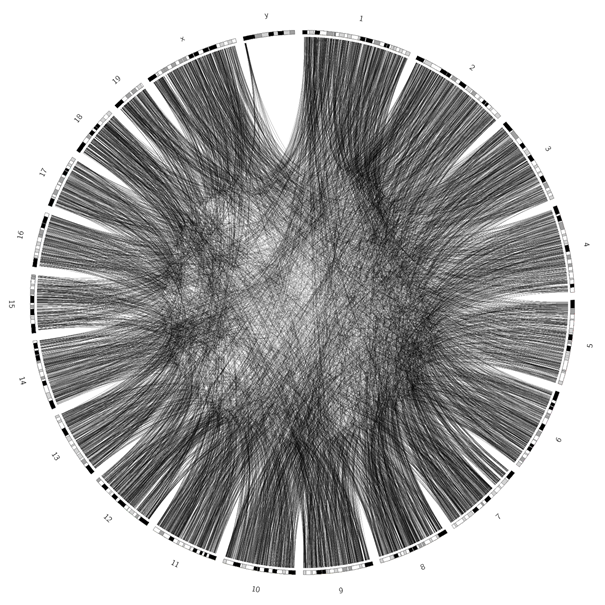

Supplement: S2 Fig — Banded ideograms represent the chromosomes of the mouse genome, black lines indicate a contact in the RMSIN. Contacts are distributed very regularly. (PNG) [file pone.0126125.s010.png]

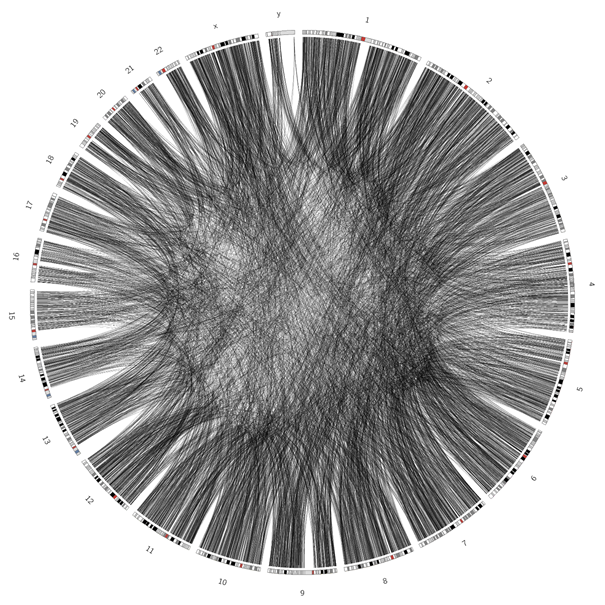

Supplement: S3 Fig — Colored ideograms represent the chromosomes of the human genome, black lines indicate a contact in the RHSIN. Contacts are distributed regularly. (PNG) [file pone.0126125.s011.png]

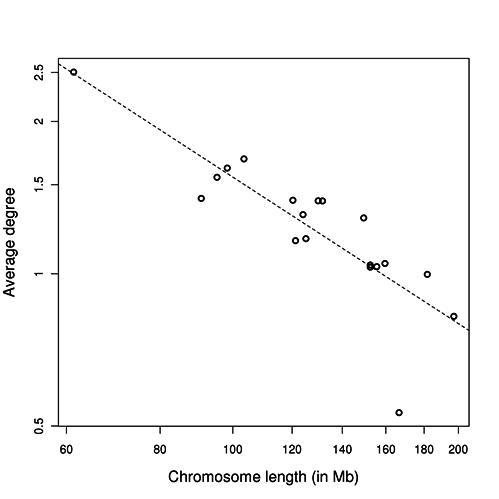

Supplement: S4 Fig — Pearson correlation coefficient -0.87. (PNG) [file pone.0126125.s012.png]

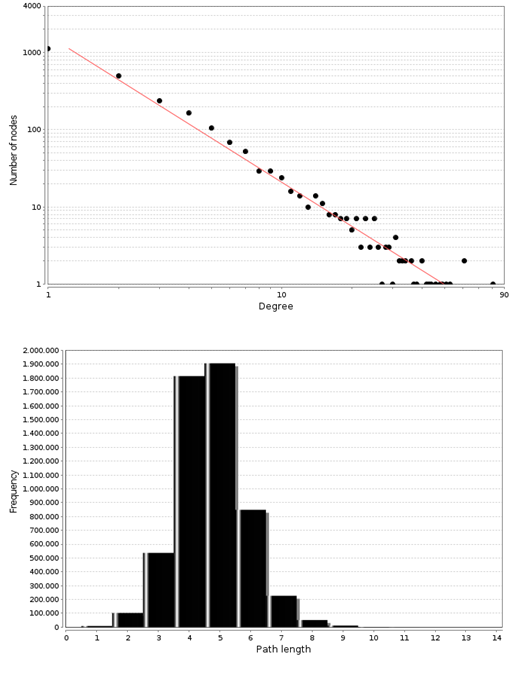

Supplement: S5 Fig — Degree distribution is shown as a log-log plot. Low degrees are very common, while higher degrees are less frequent. The red line is the fitted power law function with slope -1.896, the fit’s correlation is 0.983. Shortest path length is normally distributed and centers around a medium path length of 4.5, which is mainly caused by the existence of hubs and the power-law degree distribution. (PNG) [file pone.0126125.s013.png]

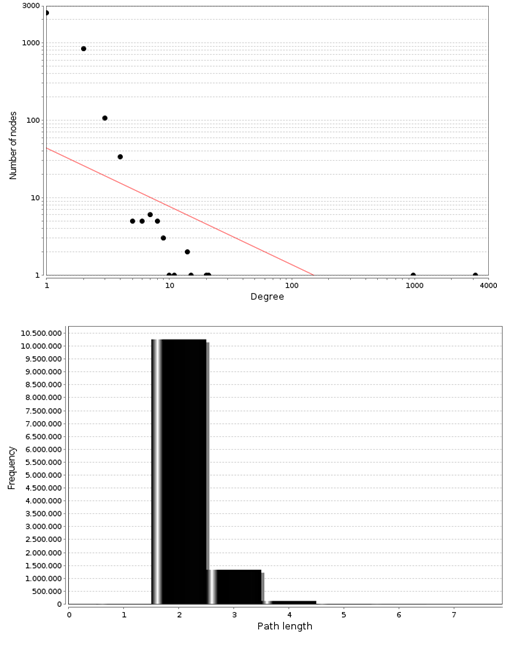

Supplement: S6 Fig — Degree distribution is shown as a log-log plot. Due to its high connectivity, (maximum) shortest path length is short and very high degrees are observable. The red line shows the fitted power law function with slope -0.752, the fit’s correlation is 0.914, though the two extreme hubs (at degrees of 1,000 and 4,000) disturb the fit. (PNG) [file pone.0126125.s014.png]

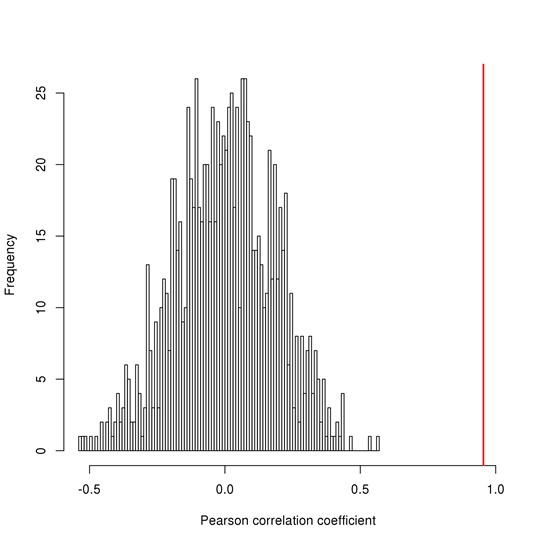

Supplement: S7 Fig — According to cumulative distribution function (CDR) observed result is significant (p-value = 0). (PNG) [file pone.0126125.s015.png]

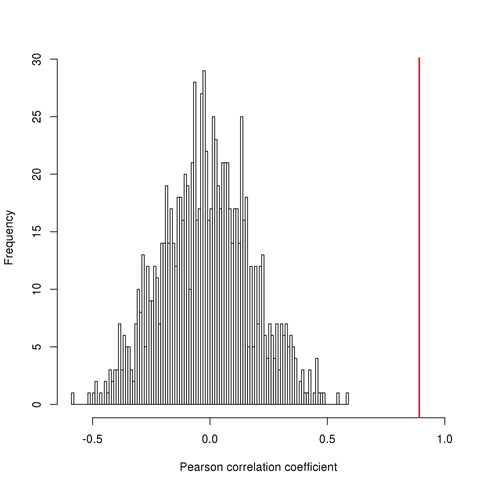

Supplement: S8 Fig — According to CDR observed result is significant (p-value = 0). (PNG) [file pone.0126125.s016.png]

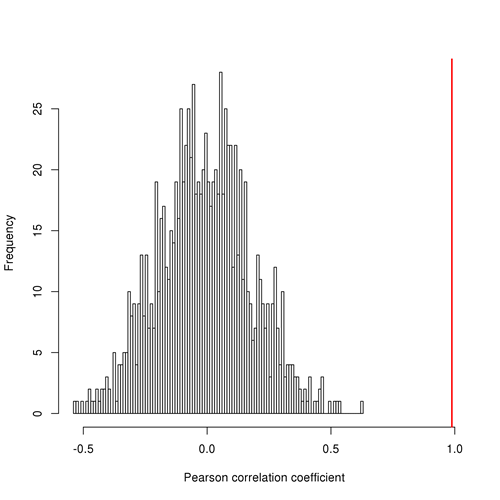

Supplement: S9 Fig — According to CDR observed result is significant (p-value = 0). (PNG) [file pone.0126125.s017.png]

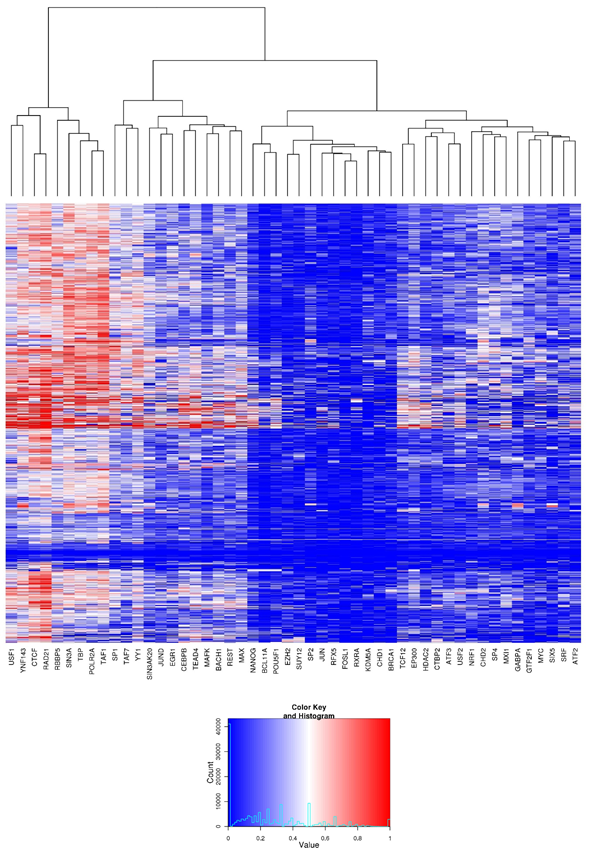

Supplement: S10 Fig — Blue color indicates no or little overlap between the genes in the cluster and the TFBS, red color indicates high overlap. There is a large section of transcription factors which rarely are involved in the genes of spatial clusters (NANOG to BRCA1), but also a set of TF that binds to these genes more often (USF1 to TAF1). CTCF, which is known to play a role in the structural organization of the genome, binds many of the genes in the clusters. (PNG) [file pone.0126125.s018.png]
